# Influence of hypertension duration and blood pressure levels on cardiovascular disease and all-cause mortality: A large prospective cohort study

**DOI:** 10.3389/fcvm.2022.948707

**Published:** 2022-10-17

**Authors:** Yan Zheng, Xiang Gao, Hai-Yi Jia, Fu-Rong Li, Hui Ye

**Affiliations:** ^1^The Fourth Affiliated Hospital Zhejiang University School of Medicine, Zhejiang, China; ^2^School of Public Health, Shanghai Jiao Tong University, Shanghai, China; ^3^Zhongshan Hospital of Fudan University, Shanghai, China; ^4^School of Public Health and Emergency Management, Southern University of Science and Technology, Shenzhen, China; ^5^Department of Epidemiology, School of Public Health, Southern Medical University, Guangzhou, China; ^6^The Second Affiliated Hospital Zhejiang University School of Medicine, Zhejiang, China

**Keywords:** hypertension duration, blood pressure, cardiovascular disease, mortality, cohort

## Abstract

**Background and objects:**

A longer duration of hypertension (HTN) has been suggested to be associated with a greater risk of cardiovascular disease (CVD). Whether such an association is similar for mortality risk, and whether HTN duration is associated with CVD/mortality beyond blood pressure (BP) control levels are yet to be assessed. We aimed to examine the associations of HTN duration and the combination of HTN duration and systolic blood pressure (SBP)/diastolic blood pressure (DBP) with risks of CVD and all-cause mortality.

**Methods:**

We used data on ∼450,000 UK residents. Participants were categorized by HTN status and HTN duration. The primary outcome was a composite of non-fatal myocardial infarction, non-fatal stroke and CVD death. We also explored the results for the above-mentioned CVD outcomes separately. All-cause mortality was also used as a secondary outcome. The age at HTN diagnosis was obtained by self-report. HTN duration was calculated as baseline age minus age at diagnosis.

**Results:**

Among all participants, compared with non-hypertensive participants, those with a longer HTN duration had increased risks of CVD and all-cause mortality. These associations persisted among hypertensive patients. Specifically, compared with patients with HTN durations of < 5 y, patients with a HTN duration of 5 to < 10 y, 10 to < 15 y, and ≥ 15 y had adjusted HRs (95% CI) of 1.09 (1.03, 1.17), 1.21 (1.13, 1.31), and 1.38 (1.29, 1.48) for composite CVD (*P*-trend < 0.001); and 1.03 (0.97, 1.08), 1.09 (1.02, 1.16), and 1.17 (1.11, 1.24) for all-cause mortality (*P*-trend < 0.001). When compared with hypertensive patients with BP < 140/90 mmHg and a HTN duration of < 5 y, adjusted HRs of CVD and all-cause mortality were 1.35 (1.15, 1.57) and 1.26 (1.11, 1.42) for those with BP < 140/90 mmHg and a duration of ≥ 15 y, and 1.43 (1.26, 1.60) and 1.13 (1.03, 1.25) for those with BP ≥ 140/90 mmHg and durations of ≥ 15 y, respectively.

**Conclusion:**

A longer HTN duration was associated with increased risks of CVD and overall death in a linear fashion, and these associations were independent of BP control levels.

## Introduction

Hypertension (HTN) is the largest single contributor to the global burden of cardiovascular disease (CVD) and mortality, leading to 9.4 million deaths each year (1). The global burden of HTN was projected to increase from 918 million adults in 2,000 to 1.56 billion in 2025 (2), reflecting an expected increase in prevalent HTN from 26.4 to 29.2% worldwide. Paralleled with the HTN epidemic is that people with HTN are living longer. As such, HTN duration, a reflector of cumulative exposure to high blood pressure (BP), may be recognized as an important factor when considering health risks associated with BP levels.

To date, mounting epidemiological evidence has demonstrated that higher levels of BP or even lower levels of BP are both associated with CVD risks and premature death among people with HTN (3–6). There are, however, a paucity of studies investigating the association between HTN duration and risk of CVD. In fact, existing evidence has been mainly focused on the effects of the age at HTN onset on CVD (7, 8), is restricted to a particularly high-risk population (e.g., patients with atrial fibrillation [AF]) (9), or explores only the links between HTN duration and heart failure (HF) (10). Thus, the associations of HTN duration with long-term CVD risks and death are far from being fully elucidated. Additionally, whether the CVD risks associated with HTN duration differ in hypertensive patients with different BP control levels is unclear.

We investigated the associations of HTN duration and BP levels with the risk of CVD and death in participants from the UK Biobank, a nationwide cohort study. We hypothesize that a longer HTN duration is associated with higher risks of CVD and death in a linear fashion. Given that cumulative exposure to long-term suboptimal BP control would result in adverse health risks, a second hypothesis we tested is that the detrimental effects of long HTN duration on CVD and death would be similar regardless of a single measurement of baseline BP control levels.

## Materials and methods

### Study population

From 2006 to 2010, the UK Biobank collected data from approximately 500,000 consenting participants (aged 37–73 y) across 22 assessment centers in the UK. At the baseline assessment, biological measurements were collected and touch-screen questionnaires were used to obtain information on sociodemographic characteristics, lifestyle, medical conditions and health status. Details of the study have been described elsewhere (11–13). We restricted our analysis to those 455,932 participants who had available data on BP measures and age at HTN diagnosis at baseline and were also free of a prior history of CVD (angina, myocardial infarction, stroke), were not lost to follow-up and did not withdraw from the study (Supplementary Figure 1).

The UK Biobank Study was approved by the National Health Service National Research Ethics Service (REC reference: 11/NW/03820), and all participants provided written informed consent to participate in the study.

### Exposures

Two BP measurements were taken while the participant was seated after 5 min of rest using an appropriate cuff and an Omron HEM-7015IT digital BP monitor (14). Mean SBP and DBP values were calculated from 2 automated (*n* = 426,049) or 2 manual (*n* = 29,883) BP measurements. We defined HTN as self-reported HTN, self-reported hypertensive medication use, or an SBP/DBP level of ≥ 140/90 mmHg. Self-reported age at hypertension onset was ascertained by the question “What was your age when the high blood pressure was first diagnosed?” Those with a SBP/DBP level of ≥ 140/90 mmHg but were not considered to have HTN was defined as undiagnosed HTN, and were assigned a duration of 0 y (*n* = 147,501).

### Assessment of the outcomes

The date and cause of hospital admission were ascertained by record linkage to Health Episode Statistics records for participants in England and Wales, and the date and cause of death were obtained from death certificates in the National Health Service Information Centre for participants from England and Wales and the National Health Service Central Register (Scotland) for participants from Scotland. The primary outcome was composite CVD: non-fatal myocardial infarction (MI), non-fatal stroke, and CVD death. The secondary outcomes were the above events assessed individually. All-cause mortality was also used as a secondary outcome, which was defined as deaths from all causes. At the time of analysis, hospital admission data were available through March 29, 2017, and mortality data were available through April 26, 2020, for England and Wales and April 20, 2020, for Scotland; these dates were used as the end of follow-up. Follow-up time was calculated from the date of baseline recruitment to the date of diagnosis of the event, death, or the end of follow-up, whichever occurred first.

The International Classification of Diseases, tenth revision (ICD-10) codes were used in death records, while ICD-10 and International Classification of Diseases, ninth revision (ICD-9) codes were used in medical records. MI was defined as ICD-9 codes 410-414 and ICD-10 codes I20-I25. Stroke was defined as ICD-9 codes 430-434 and ICD-10 codes I60-I63. CVD death was defined as ICD-10 codes I00-I99.

### Covariates

The UK Biobank used a baseline touch screen questionnaire to collect information on the following potential confounders: sociodemographic information (age, sex, and race/ethnicity); area-based social deprivation (Townsend score, an index for socioeconomic deprivation, and household income); lifestyle (smoking status, dietary habits, and physical activity); and self-reported medical conditions (medications used to treat high cholesterol or HTN; history of diabetes and longstanding illness). A non-stretchable tape was used to measure height, and a Tanita BC-418 MA body analyzer was used to measure weight (15). Body mass index (BMI) was calculated as weight in kilograms divided by height in meters squared (kg/m^2^) and was categorized according to the WHO classification scheme as follows: < 18.5 kg/m^2^ (underweight), 18.5–24.9 kg/m^2^ (normal weight), 25.0–29.9 kg/m^2^ (overweight), 30.0–34.9 kg/m^2^ (class I obesity), and 35.0 kg/m^2^ or greater (class II or III obesity). Longstanding illness was ascertained by the question “Do you have any longstanding illness, disability or infirmity?” Dietary habits were assessed through a food frequency questionnaire. A healthy diet score was calculated using the method described previously (16). Circulating lipid profiles, glycated hemoglobin (HbA1c), and C-reactive protein (CRP) were also used for analysis. Diabetes status was ascertained based on the following information: a self-reported history of diabetes or use of diabetes medications, a history of diabetes in the medical record, or a plasma HbA1c level ≥ 6.5% (48 mmol/mol). Physical activity was assessed by the International Physical Activity Questionnaire short form. We used the summed metabolic equivalents of energy (METs) per week for all activity in the analysis.

### Statistical analysis

Continuous variables are described as the means and standard deviations (SDs), and categorical variables are described by numbers and percentages (%), according to HTN status and HTN duration or BP at baseline. Participants with HTN were divided by the disease duration (< 5 y, ≥ 5 to < 10 y, ≥ 10 to < 15 y, or ≥ 15 y) or SBP/DBP levels [SBP < 140 mmHg and DBP < 90 mmHg (low BP), SBP 140–159 mmHg or DBP 90–99 mmHg (Grade 1 HTN), SBP 160–179 mmHg or DBP 100–110 mmHg (Grade 2 HTN) and SBP ≥ 180 mmHg or DBP ≥ 110 (Grade 3 HTN)]. We also categorized participants by SBP levels alone or DBP levels alone.

We applied multivariable Cox regression models to estimate the hazard ratios (HRs) and 95% confidence intervals (CIs) for the outcomes of interest after confirming the assumptions of the proportionality of hazards by examinations of Schoenfeld residuals. Models were adjusted for potential confounders that may be associated with both BP and CVD. Responses of “not known” or “prefer not to answer” to the covariates were combined into an “unknown” category. Missing values accounted for < 2.8% of all the covariates. Age, sex and other potential confounders were adjusted for, including ethnicity (white, black, South Asian, or other), Townsend score, BMI, smoking status (current/previous/never), healthy diet score, diabetes, longstanding illness, hypertensive drug use, and cholesterol-lowering medication use; HTN duration and BP (both SBP and DBP) were also mutually adjusted for each other in the respective analyses. Tests of trends were conducted by using the median value for each category of HTN duration or BP as a continuous variable in multivariate models. The statistical significance of the interactions was assessed by adding a product term into the model. We also used Cox models with penalized splines to present the associations between endpoints and HTN duration or BP on a continuous scale (17).

For the subgroup analysis, we examined the associations of HTN duration and BP categories with CVD and death according to age (< 65 y or ≥ 65 y), sex, SBP/DBP levels (< 140 mmHg/90 mmHg or ≥ 140/90 mmHg), or HTN duration (< 5 y or ≥ 5 y). Several sensitivity analyses were performed for the primary outcome by additionally adjusting for CRP, total cholesterol (CHO), high-density lipoprotein (HDL) cholesterol, physical activity, and by exclusion of those (1) missing data for covariates, (2) having composite CVD within the first 2 y of follow-up, (3) or having undiagnosed HTN. Stratified Cox proportional hazards models by adjusting for age deciles as strata were also performed to test the robustness of the main results.

Analyses were conducted using Stata version 14.0 (College Station, Texas) and R version 3.4.2 (R foundation for Statistical Computing). A *P*-value < 0.05 was considered statistically significant.

## Results

During follow-up, we identified 13,624 composite CVD events, including 6,533 non-fatal MIs, 4,135 non-fatal strokes and 4,118 CVD deaths) and a total of 23,759 all-cause deaths (median follow-up 11.2 y). The baseline characteristics of the participants are presented in Table 1. A total of 253,842 (55.7%) participants were considered to have HTN and included in the duration analysis: 74.5% had durations of < 5 y, 11.5% had durations of 5 to < 10 y, 6.5% had durations of 10 to < 15 y and 7.5% had durations of ≥ 15 y. Participants with HTN were older and more likely to be men but they tended to have higher BMI, diabetes, use of cholesterol-lowering medication and have higher rates of longstanding illness than participants without HTN. Among those with HTN, longer HTN durations were associated with lower BP levels, older age, higher BMI, and an increased likelihood to have longstanding illness but a reduced likelihood to be current smokers. The proportion of hypertensive participants’ baseline SBP/DBP levels was as follows: (1) 5.9% with low BP; (2) 32.7% with Grade 1 HTN; (3) 13.5% with Grade 2 HTN; and (4) 3.7% with Grade 3 HTN. Characteristics of participants by BP levels are shown in Supplementary Tables 1–3.

**TABLE 1:** Baseline characteristics of the included participants by hypertension status and hypertension duration.

	Without HTN	With HTN, by duration (y)			
		<5	≥5 to < 10	≥10 to < 15	≥15
No. participants	202,090	189,220	29,256	16,397	18,969
HTN duration, y	NA	0.4 (1.0)	6.8 (1.4)	11.6 (1.4)	22.8 (7.3)
SBP, mmHg	123.8 (10.0)	152.6 (14.9)	150 (18.7)	150 (19.0)	150.8 (19.8)
DBP, mmHg	75.4 (76)	88.1 (9.4)	87.1 (10.5)	86.6 (10.5)	86.4 (10.5)
Age, y	53.5 (8.0)	57.6 (7.7)	59.3 (7.0)	60.0 (6.6)	60.7 (6.7)
Men, %	74,789 (37.0)	95,158 (50.3)	15,867 (54.2)	8,788 (53.6)	8,440 (44.5)
White, %	189,401 (93.7)	179,667 (95.0)	27,550 (94.2)	15,276 (93.2)	17,758 (93.6)
Townsend score	–1.32 (3.1)	–1.45 (3.0)	–1.28 (3.1)	–1.27 (3.1)	–1.28 (3.1)
BMI, kg/m^2^	26.0 (4.2)	27.9 (4.7)	29.3 (5.1)	29.5 (5.3)	29.3 (5.3)
Smoker, %	23,437 (11.6)	18,531 (9.8)	2,604 (8.9)	1,338 (8.2)	1,370 (7.2)
Healthy diet score	2.9 (1.4)	2.9 (1.4)	2.9 (1.4)	2.9 (1.4)	3.0 (1.4)
Diabetes, %	4,964 (2.5)	9,059 (4.8)	3,761 (12.9)	2,279 (13.9)	2,450 (12.9)
Hypertensive medication use,%	NA	14,497 (7.7)	12,334 (42.2)	7,322 (44.7)	7,617 (40.2)
Cholesterol-lowering medication use, %	12,052 (6.0)	24,277 (12.8)	10,331 (35.3)	6,157 (37.6)	6,953 (36.7)
Longstanding illness, %	49,419 (24.5)	53,283 (28.2)	12,389 (42.4)	7,337 (44.8)	9,076 (47.9)

Continuous variables are described as means (standard deviations), and categorical variables are described as numbers and percentages. HTN, hypertension; SBP, systolic blood pressure; DBP, diastolic blood pressure; BMI, body mass index.

### Hypertension duration and cardiovascular disease and death

**FIGURE 1:**
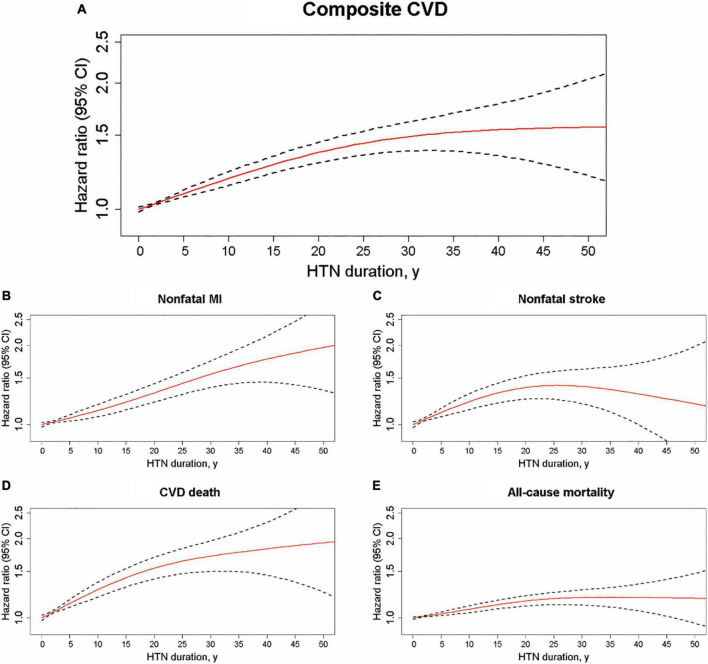
Hypertension duration on a continuous scale and risks of cardiovascular disease and all-cause mortality among participants with hypertension. Hazard ratio (solid red line) and 95% confidence interval (dashed black lines) from Cox regression using penalized splines. Multifactorial adjustments were made for age, sex, ethnicity, Townsend score, body mass index, smoking, healthy diet score, diabetes, longstanding illness, hypertensive drug use, cholesterol-lowering medication use, systolic blood pressure and diastolic blood pressure. HTN, hypertension; CVD, cardiovascular disease; MI, myocardial infarction. **(A)** HTN duration and composite CVD, **(B)** HTN duration and nonfatal MI, **(C)** HTN duration and nonfatal stroke, **(D)** HTN duration and CVD death, and **(E)** HTN duration and all-cause mortality.

Longstanding HTN resulted an increased risk of composite CVD among all participants or among those with HTN (Table 2). For participants with HTN, model 2 yielded significant results for the duration-CVD associations, and these associations remained after further adjusting for SBP and DBP in model 3. Specifically, compared with participants with HTN durations of < 5 y, the adjusted HRs (95% CI) were 1.09 (1.03, 1.17), 1.21 (1.13, 1.31), and 1.38 (1.29, 1.48) for HTN durations of 5 to < 10 y, 10 to < 15 y and ≥ 15 y (*P*-trend < 0.01), respectively. Each 5-y increase in HTN duration resulted in an adjusted HR (95% CI) of 1.07 (1.06, 1.08). We also found significantly higher risks of all the secondary outcomes (Supplementary Tables 4, 5); those with HTN durations of ≥ 15 y had adjusted HRs (95 CIs) of 1.36 (1.23, 1.50) for non-fatal MI, 1.39 (1.23, 1.58) for non-fatal stroke, 1.57 (1.40, 1.76) for CVD death, and 1.17 (1.11, 1.24) for all-cause mortality, compared with participants with HTN durations of < 5 y. When HTN duration was modeled continuously, the relationship between HTN duration and composite CVD risk was increased in a monotonic manner. The linear associations were most marked for CVD death, followed by non-fatal MI, non-fatal stroke and all-cause mortality (Figure 1).

**TABLE 2:** Hazard ratios and 95% confidence intervals for the associations between hypertension duration and risk of composite cardiovascular disease.

	Without HTN	With HTN, by duration (y)			
		<5	≥5 to < 10	≥ 10 to < 15	≥15
Person-y	1,631,170	1,509,563	231,462	128,413	148,380
Composite CVD	3,280	6,786	1,441	917	1,200
**Among all participants**					
HR1 (Age and sex adjusted)	1.0 (Ref)	1.58 (1.51, 1.65)	1.91 (1.79, 2.04)	2.16 (2.00, 2.32)	2.47 (2.31, 2.65)
HR2*	1.0 (Ref)	1.54 (1.47, 1.62)	1.56 (1.45, 1.69)	1.73 (1.58, 1.89)	2.02 (1.86, 2.20)
**Among hypertensive patients**					
HR1 (age and sex adjusted)	NA	1.0 (Ref)	1.22 (1.15, 1.30)	1.40 (1.30, 1.50)	1.58 (1.49, 1.69)
HR2*	NA	1.0 (Ref)	1.07 (1.00, 1.14)	1.21 (1.12, 1.31)	1.37 (1.28, 1.47)
HR3^†^ (model 2 + BP)	NA	1.0 (Ref)	1.09 (1.03, 1.17)	1.21 (1.13, 1.31)	1.38 (1.29, 1.48)

Ref indicates reference. Values in parentheses are 95% CIs. *Model 2 was adjusted for age, sex, ethnicity, Townsend score, body mass index, smoking status, healthy diet score, diabetes, longstanding illness, hypertensive drug use, and cholesterol-lowering medication use. ^†^Model 3 was adjusted for variables in model 2 and systolic blood pressure and diastolic blood pressure. HTN, hypertension; BP, blood pressure; NA, not applicable.

### Blood pressure and cardiovascular disease and death

Participants with higher levels of BPs generally had greater risks of CVD and premature death than their non-hypertensive counterparts (Supplementary Tables 6–8). Among participants with HTN, the fully adjusted HRs (95% CI) for the risk of composite CVD were 0.92 (0.87, 0.99) for Grade 1 HTN, 1.12 (1.05, 1.20) for Grade 2 HTN and 1.45 (1.34, 1.58) for Grade 3 HTN compared with those with low BP (Supplementary Table 9). The adjusted HRs (95%) comparing the Grade 3 HTN with the low BP groups were 1.46 (1.29, 1.65) for non-fatal MI, 1.41 (1.21, 1.64) for non-fatal stroke, 1.51 (1.31, 1.75) for CVD death and 1.15 (1.07, 1.23) for all-cause mortality. Regarding SBP or DBP alone, we observed similar patterns of associations for all outcomes (Supplementary Tables 9–11). Detailed studies of the dose-response associations indicated that J-shaped associations were found between SBP levels and DBP levels and all outcomes, especially for composite CVD, CVD death and all-cause mortality (Supplementary Figures 2, 3).

### Hypertension duration and blood pressure and cardiovascular disease and death

**FIGURE 2:**
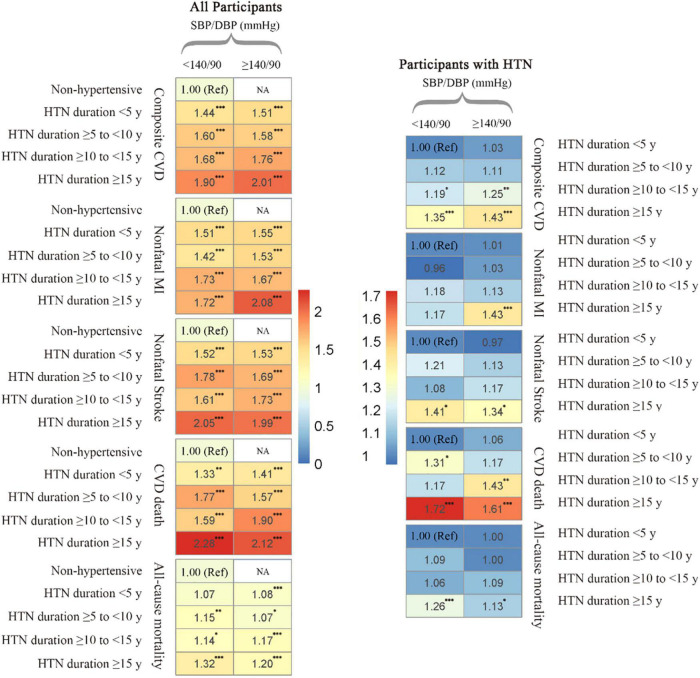
Cardiovascular disease and all-cause mortality risk matrix for the combined effect of hypotension duration and blood pressure control levels among all participants and participants with hypertension. Risk matrixes were developed using Cox models by blood pressure control levels (SBP/DBP < 140/90 or ≥ 140/90 mmHg). Models were adjusted for age, sex, ethnicity, Townsend score, body mass index, smoking, healthy diet score, diabetes, longstanding illness, hypertensive drug use, and cholesterol-lowering medication use. HTN, hypertension; CVD, cardiovascular disease; SBP, systolic blood pressure; DBP, diastolic blood pressure; MI, myocardial infarction; Ref, reference; NA, not applicable. ***P < 0.001; **P < 0.01; *P < 0.05.

When HTN duration and BP were combined, we found that regardless of BP control levels (SBP/DBP < 140/90 or ≥ 140/90 mmHg), longstanding HTN duration was associated with a higher risk of composite CVD (Figure 2). The adjusted HR for composite CVD was 1.90 for those with SBP/DBP levels of < 140/90 mmHg and HTN durations of ≥ 15 y and 2.01 for those with SBP/DBP levels of ≥ 140/90 mmHg and HTN durations of ≥ 15 y, compared with non-hypertensive participants. Compared with hypertensive participants with SBP/DBP < 140 mmHg and HTN durations of < 5 y, the corresponding adjusted HRs were 1.35 (SBP/DBP < 140/90 mmHg and HTN durations ≥ 15 y) and 1.43 (SBP/DBP ≥ 140/90 mmHg and HTN durations ≥ 15 y). We also found similar trends of hazards for non-fatal MI as composite CVD, but more pronounced duration-associations were indicated for non-fatal stroke, CVD death and all-cause mortality among those with SBP/DBP < 140/90 mmHg relative to those with SBP/DBP ≥ 140/90 mmHg. Similar patterns of combined associations were also found when HTN duration groups were combined with SBP levels alone (SBP < 140 mmHg or ≥ 140 mmHg) or DBP levels alone (DBP < 90 mmHg or ≥ 90 mmHg) (Supplementary Figures 4, 5).

### Subgroup analysis and sensitivity analysis

Subgroup analysis showed that, among all participants, the associations of HTN duration and composite CVD were more pronounced among younger adults (< 65 y) than older adults (≥ 65 y) (*P*-interaction < 0.001), and women with longstanding HTN also tended to have a higher risk than men with longstanding HTN (*P*-interaction < 0.001) (Supplementary Table 4). Interactions for age and sex were also found among hypertensive patients, and dose-response analyses showed that the hazards of composite CVD increased linearly with the increase in HTN duration among the subgroup aged < 65 y and then reached plateau in the subgroup aged ≥ 65 y. Similar trends were also observed between sexes, where a more linear relationship was observed for men than women, although the risk increased steeper initially for women (Supplementary Figure 6). Interactions regarding age and sex were also significant for the associations of SBP/DBP and SBP with composite CVD, with stronger associations and more pronounced U-shaped SBP-CVD associations indicated among younger adults and women than older adults and men (Supplementary Tables 6, 7, 9, 10 and Supplementary Figure 7). No significant interactions were found in subgroup analyses for DBP and composite CVD (Supplementary Tables 8, 11, Supplementary Figure 8). Our primary results were not materially changed in a series of sensitivity analyses (Supplementary Table 12).

## Discussion

In the UK Biobank Study of > 400,000 participants with and without HTN, we found that longer HTN duration was strongly associated with increase in the risks of CVD and all-cause mortality; furthermore, elevated BPs were also associated with higher risks of CVD and death and low BP levels also conferred greater excess risks, especially for death events. These associations for HTN duration and BP were independent of each other, and the results regarding HTN duration were robust enough in a series of sensitivity analyses. In addition, regardless of BP cutoffs (SBP/DBP < 140/90 or ≥ 140/90 mmHg), longer HTN durations were consistently associated with high risks of all endpoints. The excess risk of composite CVD associated with HTN duration and BPs was more pronounced among younger adults (<65 y) than older adults (≥65 y) and among women than men.

Over recent years, many publications have been focusing on using a series of different indices to capture the overall BP exposure; for example, time-averaged BP, cumulative BP, BP trajectory patterns, and age of hypertension onset (18). However, only a few studies have examined the question of CVD complications according to HTN duration. Kim et al. found that the increase in HTN duration was associated with an increased risk of stroke during a mean follow-up of 0.7 y, using a sample of > 240,000 AF patients in Korea (9). Another study of > 9,000,000 Korean participants found that a longer HTN duration at baseline was an important risk factor for incident AF after a mean follow-up of 8.2 y. Mefford et al. also found that a longer HTN duration was associated with a risk of incident heart failure (HF) among > 17,000 community-dwelling white and black participants (mean follow-up 8.3 y). These studies, however, were either focused on populations with AF and had a relatively short follow-up period or included AF/HF as the only outcome of interest; thus, the results may not be treated as direct evidence regarding overall CVD risks associated the HTN duration among the general population. Of note, a recent study of > 70,000 Chinese adults showed that hypertensive patients with a young age of HTN onset had a higher risk of CVD and all-cause mortality (7). Unfortunately, this study reported only the links between age at HTN diagnosis and CVD risk, and the duration-CVD associations were not elucidated, although a younger age at HTN diagnosis may be translated into a longer HTN duration among participants of the same age. Our study was able to extend this type of investigation by exploring the associations of HTN duration and the combined effects of HTN duration and BP levels with risks of different CVD events and overall death in a general Western population.

In the present study, the risk of CVD and death associated with BP appeared to be J-shaped, suggesting that not only high but also low BP levels are detrimental. Indeed, a low BP is potentially harmful and predictive of some adverse CVD outcomes (19), although these associations are likely to be biased from the higher comorbidity burden or drug interactions caused by different medications among those with low BP (6, 20). If this is the case, our results may suggest the inability of BP alone to estimate CVD burden by HTN in this observational study. Interestingly, the risk for CVD in hypertensive patients increases in a graded fashion with increases in HTN duration with a plateau at the high end, except for those younger than 65 y or men. This result suggests that in younger patients or men with HTN, a longer duration of HTN can increase the risk of overall CVD events continuously. Another important finding in this study was that regardless of BP levels (SBP/DBP cutoffs of 140/90 mmHg), longer HTN durations were consistently associated with higher risks of CVD and death. These findings may suggest that HTN duration, although is often overlooked, is likely to be an important contributor to CVD risks beyond BP control levels; thus, HTN duration in combination with BP control levels may have the potential to better stratify patients for the risks of CVD and overall death. Further study is needed to clarify the predictive utility of health risks by subdividing HTN by disease duration.

The mechanisms underlying the relationship between HTN duration and CVD risks are multifactorial. The associations between HTN and left ventricular hypertrophy, coronary calcification, and multiple target organ damage such as proteinuria are well recognized, and longstanding HTN, particularly if suboptimally controlled, could contribute to the cumulative burden of cardiovascular damage during life, consequently increasing the risk of CVD and premature death. On the other hand, some evidence has proposed that early-onset HTN is more strongly affected by hereditary susceptibility (21, 22), and several genetic loci and gene variants that are related to early-onset HTN are also associated with incident MI (23–26). A previous study of UK Biobank also reported that lifelong genetic exposure to lower SBP was associated with lower CVD risks (27). Thus, it is likely that individuals with longstanding HTN may carry some HTN-related genetic variants that would contribute to the development of CVD.

### Strengths and limitations

This study was strengthened by including a large number of general participants with HTN from a nationwide cohort with comprehensive follow-up, and the linkage with death and hospital registries ensured complete information on the outcomes and minimized the number of participants who were lost to follow-up.

Several limitations should be considered when interpreting the results. First, a large proportion of our sample in the category of < 5 y HTN duration were hypertensive patients with unknown duration, such that those with longstanding undiagnosed HTN may be misclassified into the low-risk group (HTN duration of < 5 y). However, even this was the case, it should bring the association toward the null only and thus would not significantly change the conclusion of our research; therefore, our results could only underestimate the true association. Another concern is that there may be residual confounding. Indeed, confounding by age was of particular concern since longstanding HTN is correlated with increased age. In addition to controlling for age in our multivariable analyses, we used stratified Cox models by age deciles and found that the results were unchanged. Further, most of the UK Biobank participants were White, thus more studies are needed to validate our findings among populations with other ethnicities. Finally, there was no available information on the ambulatory BP monitoring data, and analyses of BP levels in this study should be interpreted with caution.

## Conclusion

A longer HTN duration was associated with increased risks of CVD and overall death in a linear fashion, and these associations were independent of BP control levels.

## Data availability statement

The raw data supporting the conclusions of this article will be made available by the authors, without undue reservation.

## Ethics statement

The studies involving human participants were reviewed and approved by the National Health Service National Research Ethics Service. The patients/participants provided their written informed consent to participate in this study.

## Author contributions

YZ and F-RL contributed to the statistical analyses and had primary responsibility for writing the manuscript. F-RL directed the study. XG, H-YJ, and HY contributed to the data cleaning and interpretation of the data. All authors participated in critical revision of the manuscript, read, and approved the final manuscript.
