# Supplementary material for: Influence of hypertension duration and blood pressure levels on cardiovascular disease and all-cause mortality: A large prospective cohort study
Source: Front Cardiovasc Med. 2022 Oct 17;9:948707. doi: 10.3389/fcvm.2022.948707 (PMC9618611; doi:10.3389/fcvm.2022.948707)
Supplement: Supplementary file 1 [file Data_Sheet_1.docx]

**Supplement****ary materials**

**Supplementary Table S1.** Baseline characteristics of the included participants by hypertension status and systolic blood pressure/diastolic blood pressure control levels.

|  | Without HTN |  | With HTN, by SBP/DBP control levels | | | |
| --- | --- | --- | --- | --- | --- | --- |
|  |  |  | Low BP | Grade 1 HTN | Grade 2 HTN | Grade 3 HTN |
| No. participants | 202,090 |  | 26,726 | 148,974 | 61,323 | 16,819 |
| HTN duration, y | NA |  | 8.8 (8.0) | 2.5 (5.8) | 3.6 (6.8) | 4.4 (7.7) |
| SBP, mmHg | 123.8 (10.0) |  | 128.5 (8.3) | 146.8 (7.0) | 165.0 (8.3) | 186.6 (13.3) |
| DBP, mmHg | 75.4 (7.3) |  | 78.5 (6.9) | 86.1 (7.5) | 92.7 (9.3) | 99.6 (12.0) |
| Age, y | 53.5 (8.0) |  | 58.0 (7.4) | 57.5 (7.7) | 59.4 (7.1) | 60.4 (6.9) |
| Men, % | 74,789 (37.0) |  | 12,248 (45.8) | 75,559 (50.7) | 31,889 (52.0) | 8,557 (50.9) |
| White, % | 189,401 (93.7) |  | 24,877 (93.1) | 141,059 (94.7) | 58,377 (95.2) | 15,938 (94.8) |
| Townsend score | -1.3 (3.1) |  | -1.0 (3.3) | -1.4 (3.0) | -1.50 (3.0) | -1.4 (3.0) |
| BMI, kg/m^2^ | 26.0 (4.1) |  | 29.1 (5.2) | 28.0 (4.7) | 28.4 (4.9) | 28.7 (5.1) |
| Smoker, % | 23,437 (11.6) |  | 2,855 (10.7) | 14,166 (9.5) | 5,359 (8.7) | 1,463 (8.7) |
| Healthy diet score | 2.9 (1.4) |  | 2.9 (1.4) | 2.9 (1.4) | 2.9 (1.4) | 2.9 (1.4) |
| Diabetes, % | 4,964 (2.5) |  | 3,822 (14.3) | 8,960 (6.0) | 3,795 (6.2) | 972 (5.8) |
| Hypertensive medication use, % | NA |  | 10,801 (40.4) | 17,783 (11.9) | 10,233 (16.7) | 2,953 (17.6) |
| Cholesterol-lowering medication use, % | 12,052 (6.0) |  | 9,719 (36.4) | 24,168 (16.2) | 11,026 (18.0) | 2,805 (16.7) |
| Longstanding illness, % | 49,419 (24.5) |  | 12,548 (47.0) | 45,594 (30.6) | 18,908 (30.8) | 5,035 (29.9) |

Continuous variables are described as means (standard deviations), and categorical variables are described as numbers and percentages.

Abbreviations: HTN, hypertension; SBP, systolic blood pressure; DBP, diastolic blood pressure; BMI, body mass index.

**Supplementary Table S2.** Baseline characteristics of the included participants by hypertension status and systolic blood pressure control levels.

|  | Without HTN |  | With HTN, by SBP control levels (mmHg) | | | |
| --- | --- | --- | --- | --- | --- | --- |
|  |  |  | < 140 | ≥140 to <160 | ≥160 to <180 | ≥180 |
| No. participants | 202,090 |  | 41,426 | 143,112 | 54,795 | 14,509 |
| SBP, mmHg | 123.8 (10.0) |  | 130.2 (7.7) | 148.4 (5.6) | 167.4 (5.5) | 190.1 (10.1) |
| DBP, mmHg | 75.4 (7.3) |  | 83.7 (9.2) | 86.4 (8.4) | 91.9 (9.7) | 97.4 (11.4) |
| HTN duration, y | NA |  | 6.4 (7.8) | 2.6 (5.9) | 3.7 (7.0) | 4.6 (7.9) |
| Age, y | 53.5 (8.0) |  | 56.1 (7.9) | 57.8 (7.6) | 60.1 (6.8) | 61.4 (6.2) |
| Men, % | 74,789 (37.0) |  | 19,264 (46.5) | 73,605 (51.4) | 28,180 (51.4) | 7,204 (49.7) |
| White, % | 189,401 (93.7) |  | 38,422 (92.8) | 135,720 (94.8) | 52,295 (95.4) | 13,814 (95.2) |
| Townsend score | -1.3 (3.1) |  | -1.0 (3.2) | -1.5 (3.0) | -1.5 (3.0) | -1.5 (3.0) |
| BMI, kg/m^2^ | 26.0 (4.1) |  | 29.3 (5.4) | 28.0 (4.7) | 28.2 (4.7) | 28.3 (4.8) |
| Smoker, % | 23,437 (11.6) |  | 4,534 (10.9) | 13,480 (9.4) | 4,643 (8.5) | 1,186 (8.2) |
| Healthy diet score | 2.9 (1.4) |  | 2.8 (1.4) | 2.9 (1.4) | 2.9 (1.4) | 2.9 (1.4) |
| Diabetes, % | 4,964 (2.5) |  | 4,580 (11.1) | 8,639 (6.0) | 3,478 (6.4) | 852 (5.9) |
| Hypertensive medication use, % | NA |  | 12,462 (30.1) | 17,574 (12.3) | 9,136 (16.7) | 2,598 (17.9) |
| Cholesterol-lowering medication use, % | 12,052 (6.0) |  | 11,445 (27.6) | 23,538 (16.5) | 10,180 (18.6) | 2,555 (17.6) |
| Longstanding illness, % | 49,419 (24.5) |  | 17,255 (14.7) | 43,728 (30.6) | 16,793 (30.7) | 4,309 (29.7) |

Continuous variables are described as means (standard deviations), and categorical variables are described as numbers and percentages.

Abbreviations: HTN, hypertension; SBP, systolic blood pressure; DBP, diastolic blood pressure; BMI, body mass index.

**Supplementary Table S3.** Baseline characteristics of the included participants by hypertension status and diastolic blood pressure control levels.

|  | Without HTN |  | With HTN, by DBP control levels (mmHg) | | | |
| --- | --- | --- | --- | --- | --- | --- |
|  |  |  | DBP <90 | DBP ≥90 & <100 | DBP ≥100 & <110 | DBP ≥110 |
| No. participants | 202,090 |  | 143,754 | 83,575 | 22,177 | 4,336 |
| HTN duration, y | NA |  | 3.9 (7.0) | 3.0 (6.2) | 3.6 (6.6) | 3.4 (6.9) |
| SBP, mmHg | 123.8 (10.0) |  | 147.9 (14.2) | 153.8 (15.2) | 165.7 (16.2) | 179.3 (19.2) |
| DBP, mmHg | 75.4 (7.3) |  | 81.2 (6.2) | 93.6 (2.8) | 103.2 (2.7) | 114.6 (4.9) |
| Age, y | 53.5 (8.0) |  | 59.4 (7.2) | 56.7 (7.7) | 56.6 (7.6) | 55.8 (7.7) |
| Men, % | 74,789 (37.0) |  | 67,494 (47.0) | 45,188 (54.1) | 12,967 (58.5) | 2,604 (60.1) |
| White, % | 189,401 (93.7) |  | 136,901 (95.2) | 78,596 (94.0) | 20,775 (93.7) | 3,979 (91.8) |
| Townsend score | -1.3 (3.1) |  | -1.4 (3.0) | -1.4 (3.0) | -1.3 (3.1) | -1.0 (3.2) |
| BMI, kg/m^2^ | 26.0 (4.1) |  | 27.7 (4.7) | 28.8 (4.9) | 29.5 (5.2) | 30.2 (5.7) |
| Smoker, % | 23,437 (11.6) |  | 13,297 (9.3) | 7,843 (9.4) | 2,196 (9.9) | 507 (11.7) |
| Healthy diet score | 2.9 (1.4) |  | 3.0 (1.4) | 2.8 (1.4) | 2.7 (1.4) | 2.6 (1.4) |
| Diabetes, % | 4,964 (2.5) |  | 11,917 (8.3) | 4,380 (5.2) | 1,035 (4.7) | 217 (5.0) |
| Hypertensive medication use, % | NA |  | 25,192 (17.5) | 12,112 (14.5) | 3,769 (17.0) | 697 (16.1) |
| Cholesterol-lowering medication use, % | 12,052 (6.0) |  | 31,706 (22.1) | 12,579 (15.1) | 2,989 (13.5) | 444 (10.2) |
| Longstanding illness, % | 49,419 (24.5) |  | 48,919 (34.0) | 25,190 (30.1) | 6,667 (30.1) | 1,309 (30.2) |

Continuous variables are described as means (standard deviations), and categorical variables are described as numbers and percentages.

Abbreviations: HTN, hypertension; SBP, systolic blood pressure; DBP, diastolic blood pressure; BMI, body mass index.

**Supplementary Table S4.** Hazard ratios and 95% confidence intervals for the associations between hypertension duration and risk of cardiovascular disease and all-cause mortality among all participants.

|  | Without HTN |  | With HTN, by duration (y) | | | |
| --- | --- | --- | --- | --- | --- | --- |
|  |  |  | < 5 | ≥5 to <10 | ≥10 to <15 | ≥15 |
| Person-y | 1,631,170 |  | 1,509,563 | 2,31,462 | 1,28,413 | 148,380 |
| Composite CVD | 3,280 |  | 6,786 | 1,441 | 917 | 1,200 |
| HR1 | 1.0 (Ref) |  | 1.58 (1.51, 1.65) | 1.91 (1.79, 2.04) | 2.16 (2.00, 2.32) | 2.47 (2.31, 2.65) |
| HR2* | 1.0 (Ref) |  | 1.54 (1.47, 1.62) | 1.56 (1.45, 1.69) | 1.73 (1.58, 1.89) | 2.02 (1.86, 2.20) |
| BP stratification* | *P*-interaction =0.86 |  |  |  |  |  |
| SBP <140 & DBP <90 mmHg | 1.0 (Ref) |  | 1.24 (1.09, 1.41) | 1.34 (1.18, 1.53) | 1.41 (1.20, 1.64) | 1.61 (1.40, 1.86) |
| SBP≥140 or DBP ≥90 mmHg | 1.0 (Ref) |  | 1.52 (1.45, 1.59) | 1.58 (1.46, 1.71) | 1.78 (1.62, 1.94) | 2.03 (1.87, 2.21) |
| Age stratification* | *P*-interaction <0.01 |  |  |  |  |  |
| <65 y | 1.0 (Ref) |  | 1.58 (1.49, 1.67) | 1.62 (1.47, 1.78) | 1.73 (1.54, 1.94) | 2.09 (1.87, 2.33) |
| ≥65 y | 1.0 (Ref) |  | 1.40 (1.28, 1.53) | 1.41 (1.23, 1.61) | 1.66 (1.42, 1.93) | 1.88 (1.64, 2.15) |
| Sex stratification* | *P*-interaction <0.01 |  |  |  |  |  |
| Men | 1.0 (Ref) |  | 1.49 (1.40, 1.58) | 1.56 (1.42, 1.72) | 1.66 (1.49, 1.86) | 1.91 (1.72, 2.12) |
| Women | 1.0 (Ref) |  | 1.59 (1.47, 1.72) | 1.52 (1.32, 1.75) | 1.82 (1.55, 2.14) | 2.15 (1.88, 2.46) |
| Nonfatal MI |  |  |  |  |  |  |
| HR1 (age and sex adjusted) | 1.0 (Ref) |  | 1.63 (1.53, 1.73) | 1.82 (1.65, 2.01) | 2.12 (1.90, 2.38) | 2.50 (2.25, 2.77) |
| HR2* | 1.0 (Ref) |  | 1.55 (1.45, 1.65) | 1.49 (1.34, 1.66) | 1.69 (1.50, 1.91) | 2.03 (1.81, 2.27) |
| Nonfatal stroke |  |  |  |  |  |  |
| HR1 (age and sex adjusted) | 1.0 (Ref) |  | 1.55 (1.43, 1.68) | 1.90 (1.49, 2.14) | 1.92 (1.66, 2.22) | 2.28 (2.01, 2.59) |
| HR2* | 1.0 (Ref) |  | 1.52 (1.41, 1.66) | 1.70 (1.49, 1.94) | 1.70 (1.45, 1.99) | 2.00 (1.73, 2.30) |
| CVD death |  |  |  |  |  |  |
| HR1 (age and sex adjusted) | 1.0 (Ref) |  | 1.52 (1.39, 1.65) | 2.14 (1.91, 2.41) | 2.57 (2.25, 2.93) | 2.94 (2.61, 3.32) |
| HR2* | 1.0 (Ref) |  | 1.43 (1.31, 1.55) | 1.56 (1.37, 1.78) | 1.75 (1.52, 2.02) | 2.07 (1.81, 2.37) |
| All-cause mortality |  |  |  |  |  |  |
| HR1 (age and sex adjusted) | 1.0 (Ref) |  | 1.10 (1.07, 1.14) | 1.29 (1.23, 1.35) | 1.39 (1.31, 1.47) | 1.49 (1.41, 1.57) |
| HR2* | 1.0 (Ref) |  | 1.09 (1.05, 1.12) | 1.09 (1.03, 1.16) | 1.16 (1.08, 1.23) | 1.23 (1.16, 1.31) |

Ref indicates reference. Values in parentheses are 95% CIs.

*Model 2 was adjusted for age, sex, ethnicity, Townsend score, body mass index, smoking status, healthy diet score, diabetes, longstanding illness, hypertensive drug use, and cholesterol-lowering medication use.

Abbreviations: HTN, hypertension; CVD, cardiovascular disease; HR, hazard ratios.

**Supplementary Table S5.** Hazard ratios and 95% confidence intervals for the associations between hypertension duration and risk of cardiovascular disease and all-cause mortality among participants with hypertension.

|  | With HTN, by duration (y) | | | |  | Each 5 y increase |  | *P*-trend |
| --- | --- | --- | --- | --- | --- | --- | --- | --- |
|  | < 5 y | ≥5 to <10 y | ≥10 to <15 y | ≥15 |  |  |  |  |
| Person-y | 1,509,563 | 2,31,462 | 1,28,413 | 148,380 |  |  |  |  |
| Composite CVD | 6,786 | 1,441 | 917 | 1,200 |  |  |  |  |
| HR1 (age and sex adjusted) | 1.0 (Ref) | 1.22 (1.15, 1.30) | 1.40 (1.30, 1.50) | 1.58 (1.49, 1.69) |  | 1.11 (1.09, 1.12) |  | <0.001 |
| HR2* | 1.0 (Ref) | 1.07 (1.00, 1.14) | 1.21 (1.12, 1.31) | 1.37 (1.28, 1.47) |  | 1.07 (1.05, 1.08) |  | <0.001 |
| HR3† (model 2 + BP) | 1.0 (Ref) | 1.09 (1.03, 1.17) | 1.21 (1.13, 1.31) | 1.38 (1.29, 1.48) |  | 1.07 (1.06, 1.08) |  | <0.001 |
| BP stratification† | *P*-interaction =0.64 |  |  |  |  |  |  |  |
| SBP <140 & DBP <90 mmHg | 1.0 (Ref) | 1.12 (0.98, 1.30) | 1.19 (1.00, 1.40) | 1.36 (1.16, 1.5) |  | 1.07 (1.03, 1.10) |  | <0.001 |
| SBP≥140 or DBP ≥90 mmHg | 1.0 (Ref) | 1.05 (0.98, 1.13) | 1.18 (1.08, 1.29) | 1.34 (1.24, 1.45) |  | 1.06 (1.04, 1.08) |  | <0.001 |
| Age stratification† | *P*-interaction <0.01 |  |  |  |  |  |  |  |
| <65 y | 1.0 (Ref) | 1.11 (1.03, 1.20) | 1.19 (1.08, 1.31) | 1.39 (1.27, 1.53) |  | 1.08 (1.06, 1.10) |  | <0.001 |
| ≥65 y | 1.0 (Ref) | 1.07 (0.96, 1.19) | 1.27 (1.12, 1.43) | 1.39 (1.24, 1.56) |  | 1.06 (1.04, 1.09) |  | <0.001 |
| Sex stratification† | *P*-interaction =0.01 |  |  |  |  |  |  |  |
| Men | 1.0 (Ref) | 1.13 (1.05, 1.21) | 1.20 (1.10, 1.31) | 1.37 (1.26, 1.50) |  | 1.08 (1.06, 1.09) |  | <0.001 |
| Women | 1.0 (Ref) | 1.02 (0.91, 1.15) | 1.24 (1.09, 1.42) | 1.38 (1.23, 1.54) |  | 1.06 (1.04, 1.08) |  | <0.001 |
| Nonfatal MI |  |  |  |  |  |  |  |  |
| HR1 (age and sex adjusted) | 1.0 (Ref) | 1.22 (1.15, 1.30) | 1.40 (1.30, 1.50) | 1.58 (1.49, 1.69) |  | 1.11 (1.09, 1.12) |  | <0.001 |
| HR2* | 1.0 (Ref) | 1.07 (1.00, 1.14) | 1.21 (1.12, 1.31) | 1.37 (1.28, 1.47) |  | 1.07 (1.05, 1.08) |  | <0.001 |
| HR3† (model 2 + BP) | 1.0 (Ref) | 1.01 (0.92, 1.11) | 1.14 (1.02, 1.28) | 1.36 (1.23, 1.50) |  | 1.07 (1.05, 1.10) |  | <0.001 |
| Nonfatal stroke |  |  |  |  |  |  |  |  |
| HR1 (age and sex adjusted) | 1.0 (Ref) | 1.23 (1.11, 1.37) | 1.25 (1.09, 1.43) | 1.48 (1.32, 1.67) |  | 1.08 (1.05, 1.10) |  | <0.001 |
| HR2* | 1.0 (Ref) | 1.15 (1.02, 1.29) | 1.15 (0.99, 1.33) | 1.36 (1.20, 1.55) |  | 1.05 (1.03, 1.08) |  | <0.001 |
| HR3† (model 2 + BP) | 1.0 (Ref) | 1.19 (1.06, 1.33) | 1.19 (1.03, 1.37) | 1.39 (1.23, 1.58) |  | 1.06 (1.03, 1.09) |  | <0.001 |
| CVD death |  |  |  |  |  |  |  |  |
| HR1 (age and sex adjusted) | 1.0 (Ref) | 1.42 (1.28, 1.58) | 1.71 (1.51, 1.92) | 1.96 (1.76, 2.19) |  | 1.16 (1.13, 1.18) |  | <0.001 |
| HR2* | 1.0 (Ref) | 1.14 (1.02, 1.28) | 1.34 (1.18, 1.53) | 1.53 (1.36, 1.73) |  | 1.09 (1.07, 1.12) |  | <0.001 |
| HR3† (model 2 + BP) | 1.0 (Ref) | 1.16 (1.03, 1.29) | 1.32 (1.16, 1.50) | 1.57 (1.40, 1.76) |  | 1.10 (1.07, 1.12) |  | <0.001 |
| All-cause mortality |  |  |  |  |  |  |  |  |
| HR1 (age and sex adjusted) | 1.0 (Ref) | 1.02 (0.97, 1.08) | 1.09 (1.02, 1.16) | 1.16 (1.10, 1.23) |  | 1.03 (1.02, 1.04) |  | <0.001 |
| HR2* | 1.0 (Ref) | 1.02 (0.97, 1.08) | 1.09 (1.02, 1.16) | 1.16 (1.10, 1.23) |  | 1.03 (1.02, 1.04) |  | <0.001 |
| HR3† (model 2 + BP) | 1.0 (Ref) | 1.03 (0.97, 1.08) | 1.09 (1.02, 1.16) | 1.17 (1.11, 1.24) |  | 1.03 (1.02, 1.04) |  | <0.001 |

Ref indicates reference. Values in parentheses are 95% CIs.

*Model 2 was adjusted for age, sex, ethnicity, Townsend score, body mass index, smoking status, healthy diet score, diabetes, longstanding illness, hypertensive drug use, and cholesterol-lowering medication use.

†Model 3 was adjusted for variables in model 2 and systolic blood pressure and diastolic blood pressure.

Abbreviations: HTN, hypertension; BP, blood pressure.

**Supplementary Table S6.** Hazard ratios and 95% confidence intervals for the associations between systolic blood pressure and diastolic blood pressure control levels and risk of cardiovascular disease and all-cause mortality among all participants.

|  | Without HTN |  | With HTN, by SBP/DBP control levels | | | |
| --- | --- | --- | --- | --- | --- | --- |
|  |  |  | Low BP | Grade 1 HTN | Grade 2 HTN | Grade 3 HTN |
| Person-y | 1,631,170 |  | 212,045 | 1,188,520 | 485,600 | 131,651 |
| Composite CVD | 3,280 |  | 1,245 | 5,147 | 2,883 | 1,069 |
| HR1 (age and sex adjusted) | 1.0 (Ref) |  | 2.09 (1.96, 2.23) | 1.54 (1.47, 1.61) | 1.87 (1.78, 1.97) | 2.45 (2.28, 2.62) |
| HR2* | 1.0 (Ref) |  | 1.56 (1.45, 1.68) | 1.41 (1.35, 1.48) | 1.72 (1.63, 1.81) | 2.23 (2.08, 2.40) |
| HTN duration* | *P*-interaction =0.47 |  |  |  |  |  |
| <5 y | 1.0 (Ref) |  | 1.51 (1.38, 1.65) | 1.38 (1.32, 1.45) | 1.70 (1.60, 1.79) | 2.14 (1.97, 2.31) |
| ≥5 y | 1.0 (Ref) |  | 1.60 (1.42, 1.79) | 1.54 (1.39, 1.70) | 1.74 (1.55, 1.95) | 2.50 (2.16, 2.88) |
| Age stratification* | *P*-interaction =0.05 |  |  |  |  |  |
| <65 y | 1.0 (Ref) |  | 1.61 (1.47, 1.75) | 1.46 (1.38, 1.54) | 1.79 (1.68, 1.91) | 2.36 (2.15, 2.59) |
| ≥65 y | 1.0 (Ref) |  | 1.41 (1.24, 1.60) | 1.27 (1.16, 1.38) | 1.52 (1.38, 1.66) | 1.96 (1.74, 2.19) |
| Sex stratification* | *P*-interaction =0.03 |  |  |  |  |  |
| Men | 1.0 (Ref) |  | 1.58 (1.44, 1.73) | 1.40 (1.32, 1.48) | 1.66 (1.55, 1.77) | 2.11 (1.93, 2.31) |
| Women | 1.0 (Ref) |  | 1.48 (1.32, 1.67) | 1.40 (1.29, 1.51) | 1.80 (1.64, 1.96) | 2.44 (2.16, 2.76) |
| Nonfatal MI |  |  |  |  |  |  |
| HR1 (age and sex adjusted) | 1.0 (Ref) |  | 2.02 (1.83, 2.23) | 1.59 (1.49, 1.69) | 1.88 (1.75, 2.03) | 2.40 (2.16, 2.66) |
| HR2* | 1.0 (Ref) |  | 1.53 (1.38, 1.70) | 1.46 (1.36, 1.56) | 1.72 (1.59, 1.86) | 2.18 (1.96, 2.43) |
| Nonfatal stroke |  |  |  |  |  |  |
| HR1 (age and sex adjusted) | 1.0 (Ref) |  | 2.03 (1.80, 2.29) | 1.48 (1.37, 1.61) | 1.81 (1.65, 1.99) | 2.44 (2.16, 2.77) |
| HR2* | 1.0 (Ref) |  | 1.70 (1.49, 1.93) | 1.43 (1.32, 1.56) | 1.76 (1.59, 1.93) | 2.36 (2.08, 2.68) |
| CVD death |  |  |  |  |  |  |
| HR1 (age and sex adjusted) | 1.0 (Ref) |  | 2.44 (2.17, 2.74) | 1.53 (1.40, 1.66) | 1.90 (1.73, 2.09) | 2.67 (2.36, 3.02) |
| HR2* | 1.0 (Ref) |  | 1.55 (1.37, 1.76) | 1.33 (1.22, 1.45) | 1.66 (1.51, 1.84) | 2.32 (2.05, 2.64) |
| All-cause mortality |  |  |  |  |  |  |
| HR1 (age and sex adjusted) | 1.0 (Ref) |  | 1.46 (1.39, 1.53) | 1.11 (1.07, 1.14) | 1.18 (1.14, 1.23) | 1.38 (1.30, 1.46) |
| HR2* | 1.0 (Ref) |  | 1.16 (1.10, 1.23) | 1.06 (1.03, 1.10) | 1.14 (1.10, 1.19) | 1.33 (1.26, 1.41) |

Ref indicates reference. Values in parentheses are 95% CIs.

*Model 2 was adjusted for age, sex, ethnicity, Townsend score, body mass index, smoking status, healthy diet score, diabetes, longstanding illness, hypertensive drug use, and cholesterol-lowering medication use.

Abbreviations: HTN, hypertension; CVD, cardiovascular disease; HR, hazard ratios.

**Supplementary Table S7.** Hazard ratios and 95% confidence intervals for the associations between systolic blood pressure control levels and risk of cardiovascular disease and all-cause mortality among all participants.

|  | Without HTN |  | With HTN, by SBP control levels (mmHg) | | | |
| --- | --- | --- | --- | --- | --- | --- |
|  |  |  | < 140 | ≥140 to <160 | ≥160 to <180 | ≥180 |
| Person-y | 1,631,170 |  | 331,229 | 1,140,621 | 432,706 | 113,262 |
| Composite CVD | 3,280 |  | 1,648 | 5,064 | 2,688 | 944 |
| HR1 (age and sex adjusted) | 1.0 (Ref) |  | 1.96 (1.85, 2.09) | 1.53 (1.46, 1.61) | 1.88 (1.78, 1.98) | 2.41 (2.23, 2.60) |
| HR2* | 1.0 (Ref) |  | 1.53 (1.42, 1.64) | 1.44 (1.37, 1.52) | 1.76 (1.66, 1.87) | 2.25 (2.07, 2.45) |
| HTN duration* | P-interaction =0.43 |  |  |  |  |  |
| <5 y | 1.0 (Ref) |  | 1.47 (1.35, 1.59) | 1.41 (1.34, 1.49) | 1.74 (1.63, 1.85) | 2.12 (1.93, 2.33) |
| ≥5 y | 1.0 (Ref) |  | 1.57 (1.38, 1.77) | 1.50 (1.34, 1.68) | 1.73 (1.52, 1.96) | 2.53 (2.15, 2.98) |
| Age stratification* | P-interaction <0.001 |  |  |  |  |  |
| <65 y | 1.0 (Ref) |  | 1.56 (1.44, 1.70) | 1.47 (1.39, 1.56) | 1.86 (1.72, 2.00) | 2.36 (2.11, 2.63) |
| ≥65 y | 1.0 (Ref) |  | 1.36 (1.18, 1.56) | 1.31 (1.19, 1.44) | 1.53 (1.38, 1.70) | 1.98 (1.74, 2.26) |
| Sex stratification* | P-interaction =0.01 |  |  |  |  |  |
| Men | 1.0 (Ref) |  | 1.48 (1.35, 1.62) | 1.41 (1.32, 1.50) | 1.67 (1.55, 1.80) | 2.11 (1.90, 2.34) |
| Women | 1.0 (Ref) |  | 1.56 (1.39, 1.76) | 1.44 (1.33, 1.57) | 1.90 (1.72, 2.10) | 2.50 (2.18, 2.87) |
| Nonfatal MI |  |  |  |  |  |  |
| HR1 (age and sex adjusted) | 1.0 (Ref) |  | 1.85 (1.69, 2.02) | 1.59 (1.48, 1.70) | 1.90 (1.75, 2.06) | 2.43 (2.18, 2.72) |
| HR2* | 1.0 (Ref) |  | 1.46 (1.32, 1.61) | 1.47 (1.37, 1.57) | 1.75 (1.61, 1.90) | 2.24 (2.00, 2.51) |
| Nonfatal stroke |  |  |  |  |  |  |
| HR1 (age and sex adjusted) | 1.0 (Ref) |  | 1.98 (1.78, 2.21) | 1.45 (1.34, 1.58) | 1.81 (1.64, 1.99) | 2.37 (2.07, 2.72) |
| HR2* | 1.0 (Ref) |  | 1.64 (1.46, 1.84) | 1.40 (1.28, 1.53) | 1.74 (1.57, 1.93) | 2.29 (2.00, 2.63) |
| CVD death |  |  |  |  |  |  |
| HR1 (age and sex adjusted) | 1.0 (Ref) |  | 2.25 (2.01, 2.51) | 1.53 (1.40, 1.67) | 1.84 (1.66, 2.03) | 2.59 (2.27, 2.97) |
| HR2* | 1.0 (Ref) |  | 1.46 (1.30, 1.63) | 1.34 (1.22, 1.47) | 1.62 (1.46, 1.79) | 2.29 (2.00, 2.63) |
| All-cause mortality |  |  |  |  |  |  |
| HR1 (age and sex adjusted) | 1.0 (Ref) |  | 1.38 (1.32, 1.44) | 1.11 (1.07, 1.15) | 1.15 (1.11, 1.20) | 1.34 (1.26, 1.42) |
| HR2* | 1.0 (Ref) |  | 1.12 (1.07, 1.18) | 1.06 (1.03, 1.10) | 1.12 (1.07, 1.17) | 1.30 (1.22, 1.38) |

Ref indicates reference. Values in parentheses are 95% CIs.

*Model 2 was adjusted for age, sex, ethnicity, Townsend score, body mass index, smoking status, healthy diet score, diabetes, longstanding illness, hypertensive drug use, and cholesterol-lowering medication use.

Abbreviations: HTN, hypertension; CVD, cardiovascular disease; HR, hazard ratios.

**Supplementary Table S8.** Hazard ratios and 95% confidence intervals for the associations between diastolic blood pressure control levels and risk of cardiovascular disease and all-cause mortality among all participants.

|  | Without HTN |  | With HTN, by DBP control levels (mmHg) | | | |
| --- | --- | --- | --- | --- | --- | --- |
|  |  |  | <90 | ≥90 & <100 | ≥100 & <110 | ≥110 |
| Person-y | 1,631,170 |  | 1,139,298 | 667,624 | 176,777 | 34,117 |
| Composite CVD | 3,280 |  | 5,887 | 3,121 | 1,048 | 288 |
| HR1 (age and sex adjusted) | 1.0 (Ref) |  | 1.68 (1.61, 1.76) | 1.67 (1.59, 1.76) | 2.07 (1.93, 2.22) | 3.10 (2.74, 3.49) |
| HR2* | 1.0 (Ref) |  | 1.48 (1.41, 1.55) | 1.53 (1.46, 1.61) | 1.89 (1.76, 2.03) | 2.72 (2.41, 3.07) |
| HTN duration* | *P*-interaction =0.07 |  |  |  |  |  |
| <5 y | 1.0 (Ref) |  | 1.42 (1.36, 1.49) | 1.52 (1.44, 1.60) | 1.90 (1.76, 2.05) | 2.67 (2.33, 3.05) |
| ≥5 y | 1.0 (Ref) |  | 1.66 (1.51) | 1.62 (1.44, 1.81) | 1.82 (1.55, 2.15) | 3.00 (2.29, 3.94) |
| Age stratification* | *P*-interaction =0.45 |  |  |  |  |  |
| <65 y | 1.0 (Ref) |  | 1.50 (1.41, 1.58) | 1.59 (1.50, 1.69) | 1.93 (1.78, 2.11) | 2.84 (2.46, 3.26) |
| ≥65 y | 1.0 (Ref) |  | 1.38 (1.27, 1.50) | 1.37 (1.24, 1.51) | 1.73 (1.51, 1.98) | 2.39 (1.87, 3.05) |
| Sex stratification* | *P*-interaction =0.99 |  |  |  |  |  |
| Men | 1.0 (Ref) |  | 1.45 (1.37, 1.54) | 1.49 (1.40, 1.59) | 1.78 (1.63, 1.94) | 2.66 (2.31, 3.06) |
| Women | 1.0 (Ref) |  | 1.47 (1.36, 1.59) | 1.59 (1.45, 1.73) | 2.12 (1.86, 2.42) | 2.74 (2.15, 3.51) |
| Nonfatal MI |  |  |  |  |  |  |
| HR1 (age and sex adjusted) | 1.0 (Ref) |  | 1.69 (1.59, 1.80) | 1.74 (1.62, 1.86) | 2.04 (1.84, 2.26) | 2.59 (2.15, 3.12) |
| HR2* | 1.0 (Ref) |  | 1.50 (1.40, 1.60) | 1.59 (1.48, 1.71) | 1.86 (1.67, 2.06) | 2.28 (1.89, 2.75) |
| Nonfatal stroke |  |  |  |  |  |  |
| HR1 (age and sex adjusted) | 1.0 (Ref) |  | 1.62 (1.50, 1.75) | 1.63 (1.49, 1.79) | 2.06 (1.81, 2.34) | 3.27 (2.63, 4.06) |
| HR2* | 1.0 (Ref) |  | 1.50 (1.38, 1.63) | 1.58 (1.44, 1.74) | 1.99 (1.75, 2.27) | 3.06 (2.46, 3.81) |
| CVD death |  |  |  |  |  |  |
| HR1 (age and sex adjusted) | 1.0 (Ref) |  | 1.79 (1.65, 1.94) | 1.57 (1.43, 1.72) | 2.29 (2.02, 2.60) | 3.80 (3.09, 4.66) |
| HR2* | 1.0 (Ref) |  | 1.45 (1.33, 1.58) | 1.37 (1.24, 1.51) | 2.00 (1.76, 2.28) | 3.13 (2.54, 3.84) |
| All-cause mortality |  |  |  |  |  |  |
| HR1 (age and sex adjusted) | 1.0 (Ref) |  | 1.20 (1.16, 1.24) | 1.10 (1.06, 1.14) | 1.28 (1.21, 1.36) | 1.63 (1.46, 1.81) |
| HR2* | 1.0 (Ref) |  | 1.10 (1.07, 1.14) | 1.07 (1.03, 1.11) | 1.24 (1.17, 1.32) | 1.50 (1.34, 1.67) |

Ref indicates reference. Values in parentheses are 95% CIs.

*Model 2 was adjusted for age, sex, ethnicity, Townsend score, body mass index, smoking status, healthy diet score, diabetes, longstanding illness, hypertensive drug use, and cholesterol-lowering medication use.

Abbreviations: HTN, hypertension; CVD, cardiovascular disease; HR, hazard ratios.

**Supplementary Table S9.** Hazard ratios and 95% confidence intervals for the associations between systolic blood pressure and diastolic blood pressure control levels and risk of cardiovascular disease and all-cause mortality among participants with hypertension.

|  | With HTN, by SBP/DBP control levels | | | |
| --- | --- | --- | --- | --- |
|  | Low BP | Grade 1 HTN | Grade 2 HTN | Grade 3 HTN |
| Person-y | 212,045 | 1,188,520 | 485,600 | 131,651 |
| Composite CVD | 1,245 | 5,147 | 2,883 | 1,069 |
| HR1 (age and sex adjusted) | 1.0 (Ref) | 0.73 (0.69, 0.78) | 0.90 (0.84, 0.96) | 1.18 (1.09, 1.28) |
| HR2* | 1.0 (Ref) | 0.89 (0.84, 0.95) | 1.09 (1.02, 1.17) | 1.43 (1.31, 1.55) |
| HR3† (model 2 + HTN duration) | 1.0 (Ref) | 0.92 (0.87, 0.99) | 1.12 (1.05, 1.20) | 1.45 (1.34, 1.58) |
| HTN duration† | *P*-interaction =0.45 |  |  |  |
| <5 y | 1.0 (Ref) | 0.94 (0.83, 1.05) | 1.17 (1.04, 1.32) | 1.45 (1.27, 1.66) |
| ≥5 y | 1.0 (Ref) | 0.94 (0.86, 1.02) | 1.06 (0.96, 1.16) | 1.50 (1.33, 1.69) |
| Age stratification† | *P*-interaction =0.05 |  |  |  |
| <65 y | 1.0 (Ref) | 0.93 (0.86, 1.01) | 1.14 (1.05, 1.25) | 1.49 (1.34, 1.66) |
| ≥65 y | 1.0 (Ref) | 0.92 (0.83, 1.03) | 1.09 (0.98, 1.23) | 1.40 (1.22, 1.60) |
| Sex stratification† | *P*-interaction =0.03 |  |  |  |
| Men | 1.0 (Ref) | 0.91 (0.84, 0.98) | 1.07 (0.99, 1.17) | 1.36 (1.23, 1.51) |
| Women | 1.0 (Ref) | 0.95 (0.85, 1.07) | 1.23 (1.09, 1.38) | 1.65 (1.43, 1.91) |
| Nonfatal MI |  |  |  |  |
| HR1 (age and sex adjusted) | 1.0 (Ref) | 0.78 (0.72, 0.86) | 0.94 (0.85, 1.04) | 1.20 (1.07, 1.36) |
| HR2* | 1.0 (Ref) | 0.94 (0.86, 1.03) | 1.12 (1.01, 1.24) | 1.43 (1.26, 1.62) |
| HR3† (model 2 + HTN duration) | 1.0 (Ref) | 0.98 (0.89, 1.08) | 1.16 (1.05, 1.28) | 1.46 (1.29, 1.65) |
| Nonfatal stroke |  |  |  |  |
| HR1 (age and sex adjusted) | 1.0 (Ref) | 0.73 (0.65, 0.82) | 0.90 (0.79, 1.01) | 1.21 (1.05, 1.40) |
| HR2* | 1.0 (Ref) | 0.83 (0.74, 0.94) | 1.03 (0.91, 1.17) | 1.39 (1.19, 1.61) |
| HR3† (model 2 + HTN duration) | 1.0 (Ref) | 0.86 (0.76, 0.97) | 1.05 (0.93, 1.20) | 1.41 (1.21, 1.64) |
| CVD death |  |  |  |  |
| HR1 (age and sex adjusted) | 1.0 (Ref) | 0.62 (0.56, 0.69) | 0.78 (0.70, 0.88) | 1.10 (0.96, 1.27) |
| HR2* | 1.0 (Ref) | 0.84 (0.75, 0.94) | 1.06 (0.94, 1.19) | 1.48 (1.28, 1.71) |
| HR3† (model 2 + HTN duration) | 1.0 (Ref) | 0.88 (0.79, 0.99) | 1.10 (0.97, 1.23) | 1.51 (1.31, 1.75) |
| All-cause mortality |  |  |  |  |
| HR1 (age and sex adjusted) | 1.0 (Ref) | 0.76 (0.72, 0.79) | 0.81 (0.77, 0.85) | 0.95 (0.89, 1.01) |
| HR2* | 1.0 (Ref) | 0.91 (0.86, 0.95) | 0.98 (0.93, 1.03) | 1.14 (1.06, 1.22) |
| HR3† (model 2 + HTN duration) | 1.0 (Ref) | 0.92 (0.87, 0.97) | 0.99 (0.94, 1.05) | 1.15 (1.07, 1.23) |

Ref indicates reference. Values in parentheses are 95% CIs.

*Model 2 was adjusted for age, sex, ethnicity, Townsend score, body mass index, smoking status, healthy diet score, diabetes, longstanding illness, hypertensive drug use, and cholesterol-lowering medication use.

†Model 3 was adjusted for variables in model 2 and systolic blood pressure and diastolic blood pressure.

Abbreviations: HTN, hypertension; BP, blood pressure.

**Supplementary Table S10.** Hazard ratios and 95% confidence intervals for the associations between systolic blood pressure control levels and risk of cardiovascular disease and all-cause mortality among participants with HTN.

|  | With HTN, by SBP control levels (mmHg) | | | |  | Each 10 mmHg increase |  | *P*-trend |
| --- | --- | --- | --- | --- | --- | --- | --- | --- |
|  | < 140 | ≥140 to <160 | ≥160 to <180 | ≥180 |  |  |  |  |
| Person-y | 331,229 | 1,140,621 | 432,706 | 113,262 |  | NA |  | NA |
| Composite CVD | 1,648 | 5,064 | 2,688 | 944 |  | NA |  | NA |
| HR1 (age and sex adjusted) | 1.0 (Ref) | 0.80 (0.74, 0.83) | 0.97 (0.91, 1.04) | 1.26 (1.15, 1.36) |  | 1.06 (1.04, 1.07) |  | <0.001 |
| HR2* | 1.0 (Ref) | 0.92 (0.87, 0.98) | 1.14 (1.07, 1.22) | 1.47 (1.35, 1.60) |  | 1.08 (1.07, 1.09) |  | <0.001 |
| HR3† (model 2 + HTN duration) | 1.0 (Ref) | 0.97 (0.91, 1.02) | 1.19 (1.11, 1.26) | 1.50 (1.38, 1.62) |  | 1.08 (1.07, 1.09) |  | <0.001 |
| HTN duration† | *P*-interaction =0.34 |  |  |  |  |  |  |  |
| <5 y | 1.0 (Ref) | 0.98 (0.90, 1.07) | 1.25 (1.14, 1.37) | 1.48 (1.32, 1.66) |  | 1.10 (1.08, 1.11) |  | <0.001 |
| ≥5 y | 1.0 (Ref) | 0.96 (0.89, 1.05) | 1.09 (1.00, 1.20) | 1.56 (1.38, 1.76) |  | 1.06 (1.04, 1.09) |  | <0.001 |
| Age stratification† | *P*-interaction <0.001 |  |  |  |  |  |  |  |
| <65 y | 1.0 (Ref) | 0.96 (0.89, 1.04) | 1.22 (1.12, 1.33) | 1.54 (1.37, 1.73) |  | 1.09 (1.07, 1.10) |  | <0.001 |
| ≥65 y | 1.0 (Ref) | 0.98 (0.87, 1.10) | 1.14 (1.00, 1.29) | 1.46 (1.26, 1.69) |  | 1.07 (1.05, 1.10) |  | <0.001 |
| Sex stratification† | *P*-interaction =0.02 |  |  |  |  |  |  |  |
| Men | 1.0 (Ref) | 0.97 (0.90, 1.05) | 1.15 (1.06, 1.26) | 1.45 (1.29, 1.62) |  | 1.08 (1.06, 1.09) |  | <0.001 |
| Women | 1.0 (Ref) | 0.94 (0.84, 1.05) | 1.24 (1.10, 1.40) | 1.61 (1.38, 1.88) |  | 1.09 (1.07, 1.12) |  | <0.001 |
| Nonfatal MI |  |  |  |  |  |  |  |  |
| HR1 (age and sex adjusted) | 1.0 (Ref) | 0.87 (0.80, 0.94) | 1.05 (0.96, 1.16) | 1.36 (1.20, 1.53) |  | 1.07 (1.05, 1.09) |  | <0.001 |
| HR2* | 1.0 (Ref) | 1.01 (0.92, 1.10) | 1.22 (1.11, 1.34) | 1.57 (1.39, 1.78) |  | 1.09 (1.07, 1.11) |  | <0.001 |
| HR3† (model 2 + HTN duration) | 1.0 (Ref) | 1.04 (0.95, 1.13) | 1.24 (1.13, 1.37) | 1.54 (1.36, 1.74) |  | 1.09 (1.07, 1.11) |  | <0.001 |
| Nonfatal stroke |  |  |  |  |  |  |  |  |
| HR1 (age and sex adjusted) | 1.0 (Ref) | 0.74 (0.66, 0.82) | 0.92 (0.82, 1.03) | 1.21 (1.04, 1.41) |  | 1.06 (1.04, 1.08) |  | <0.001 |
| HR2* | 1.0 (Ref) | 0.85 (0.77, 0.94) | 1.07 (0.96, 1.21) | 1.40 (1.21, 1.62) |  | 1.08 (1.05, 1.10) |  | <0.001 |
| HR3† (model 2 + HTN duration) | 1.0 (Ref) | 0.87 (0.78, 0.97) | 1.09 (1.22, 1.22) | 1.41 (1.22, 1.63) |  | 1.08 (1.05, 1.10) |  | <0.001 |
| CVD death |  |  |  |  |  |  |  |  |
| HR1 (age and sex adjusted) | 1.0 (Ref) | 0.68 (0.62, 0.76) | 0.82 (0.74, 0.92) | 1.17 (1.01, 1.35) |  | 1.04 (1.01, 1.06) |  | <0.001 |
| HR2* | 1.0 (Ref) | 0.91 (0.82, 1.00) | 1.12 (1.00, 1.25) | 1.56 (1.35, 1.79) |  | 1.07 (1.05, 1.09) |  | <0.001 |
| HR3† (model 2 + HTN duration) | 1.0 (Ref) | 0.94 (0.85, 1.04) | 1.15 (1.03, 1.28) | 1.57 (1.37, 1.81) |  | 1.07 (1.05, 1.09) |  | <0.001 |
| All-cause mortality |  |  |  |  |  |  |  |  |
| HR1 (age and sex adjusted) | 1.0 (Ref) | 0.81 (0.77, 0.84) | 0.86 (0.81, 0.90) | 1.00 (0.94, 1.07) |  | 1.00 (0.99, 1.01) |  | 0.78 |
| HR2* | 1.0 (Ref) | 0.94 (0.90, 0.99) | 1.00 (0.95, 1.06) | 1.18 (1.10, 1.26) |  | 1.02 (1.01, 1.03) |  | <0.001 |
| HR3† (model 2 + HTN duration) | 1.0 (Ref) | 0.95 (0.91, 1.00) | 1.00 (0.95, 1.06) | 1.16 (1.08, 1.25) |  | 1.02 (1.01, 1.03) |  | <0.001 |

Ref indicates reference. Values in parentheses are 95% CIs.

*Model 2 was adjusted for age, sex, ethnicity, Townsend score, body mass index, smoking status, healthy diet score, diabetes, longstanding illness, hypertensive drug use, and cholesterol-lowering medication use.

†Model 3 was adjusted for variables in model 2 and systolic blood pressure and diastolic blood pressure.

Abbreviations: HTN, hypertension; BP, blood pressure; NA, not applicable.

**Supplementary Table S11.** Hazard ratios and 95% confidence intervals for the associations between diastolic blood pressure control levels and risk of cardiovascular disease and all-cause mortality among participants with hypertension.

|  | With HTN, by DBP control levels (mmHg) | | | |  | Each 5 mmHg increase |  | *P*-trend |
| --- | --- | --- | --- | --- | --- | --- | --- | --- |
|  | DBP <90 | DBP ≥90 & <100 | DBP ≥100 & <110 | DBP ≥110 |  |  |  |  |
| Person-y | 1,139,298 | 667,624 | 176,777 | 34,117 |  | NA |  | NA |
| Composite CVD | 5,887 | 3,121 | 1,048 | 288 |  | NA |  | NA |
| HR1 (age and sex adjusted) | 1.0 (Ref) | 0.99 (0.95, 1.03) | 1.22 (1.14, 1.31) | 1.82 (1.62, 2.05) |  | 1.02 (1.01, 1.03) |  | <0.001 |
| HR2* | 1.0 (Ref) | 1.03 (0.99, 1.08) | 1.27 (1.19, 1.36) | 1.83 (1.63, 2.07) |  | 1.04 (1.02, 1.05) |  | <0.001 |
| HR3† (model 2 + HTN duration) | 1.0 (Ref) | 1.03 (0.99, 1.08) | 1.27 (1.18, 1.35) | 1.81 (1.61, 2.04) |  | 1.04 (1.02, 1.05) |  | <0.001 |
| HTN duration† | *P*-interaction =0.05 |  |  |  |  |  |  |  |
| <5 y | 1.0 (Ref) | 1.07 (1.02, 1.13) | 1.32 (1.22, 1.44) | 1.86 (1.60, 2.16() |  | 1.05 (1.04, 1.07) |  | <0.001 |
| ≥5 y | 1.0 (Ref) | 0.96 (0.89, 1.04) | 1.16 (1.03, 1.30) | 1.72 (1.40, 2.10) |  | 1.01 (0.99, 1.02) |  | 0.04 |
| Age stratification† | *P*-interaction =0.44 |  |  |  |  |  |  |  |
| <65 y | 1.0 (Ref) | 1.06 (1.01, 1.12) | 1.28 (1.18, 1.39) | 1.86 (1.62, 2.14) |  | 1.05 (1.03, 1.06) |  | <0.001 |
| ≥65 y | 1.0 (Ref) | 0.99 (0.91, 1.06) | 1.24 (1.10, 1.40) | 1.71 (1.35, 2.17) |  | 1.02 (1.00, 1.03) |  | <0.001 |
| Sex stratification† | *P*-interaction =0.94 |  |  |  |  |  |  |  |
| Men | 1.0 (Ref) | 1.02 (0.97, 1.07) | 1.20 (1.11, 1.30) | 1.79 (1.56, 2.05) |  | 1.04 (1.02, 1.05) |  | <0.001 |
| Women | 1.0 (Ref) | 1.07 (0.99, 1.16) | 1.42 (1.26, 1.61) | 1.82 (1.43, 2.32) |  | 1.03 (1.02, 1.05) |  | <0.001 |
| Nonfatal MI |  |  |  |  |  |  |  |  |
| HR1 (age and sex adjusted) | 1.0 (Ref) | 1.02 (0.96, 1.08) | 1.20 (1.09, 1.32) | 1.51 (1.26, 1.82) |  | 1.02 (1.00, 1.03) |  | <0.001 |
| HR2* | 1.0 (Ref) | 1.05 (0.99, 1.12) | 1.23 (1.12, 1.35) | 1.51 (1.25, 1.81) |  | 1.03 (1.02, 1.05) |  | <0.001 |
| HR3† (model 2 + HTN duration) | 1.0 (Ref) | 1.05 (0.99, 1.12) | 1.22 (1.11, 1.35) | 1.49 (1.24, 1.79) |  | 1.03 (1.02, 1.06) |  | <0.001 |
| Nonfatal stroke |  |  |  |  |  |  |  |  |
| HR1 (age and sex adjusted) | 1.0 (Ref) | 1.00 (0.93, 1.09) | 1.27 (1.12, 1.43) | 2.01 (1.62, 2.48) |  | 1.03 (1.01, 1.05) |  | <0.001 |
| HR2* | 1.0 (Ref) | 1.05 (0.97, 1.14) | 1.33 (1.18, 1.50) | 2.05 (1.65, 2.53) |  | 1.05 (1.03, 1.07) |  | <0.001 |
| HR3† (model 2 + HTN duration) | 1.0 (Ref) | 1.05 (0.97, 1.14) | 1.32 (1.17, 1.49) | 2.02 (1.63, 2.51) |  | 1.05 (1.03, 1.07) |  | <0.001 |
| CVD death |  |  |  |  |  |  |  |  |
| HR1 (age and sex adjusted) | 1.0 (Ref) | 0.87 (0.80, 0.94) | 1.27 (1.13, 1.43) | 2.10 (1.72, 2.56) |  | 1.00 (0.98, 1.01) |  | <0.001 |
| HR2* | 1.0 (Ref) | 0.93 (0.86, 1.01) | 1.37 (1.22, 1.54) | 2.14 (1.75, 2.61) |  | 1.02 (1.00, 1.04) |  | <0.001 |
| HR3† (model 2 + HTN duration) | 1.0 (Ref) | 0.94 (0.86, 1.02) | 1.36 (1.21, 1.53) | 2.11 (1.73, 2.58) |  | 1.02 (1.00, 1.04) |  | <0.001 |
| All-cause mortality |  |  |  |  |  |  |  |  |
| HR1 (age and sex adjusted) | 1.0 (Ref) | 0.91 (0.88, 0.94) | 1.06 (1.00, 1.12) | 1.34 (1.20, 1.49) |  | 0.98 (0.97, 0.99) |  | 0.10 |
| HR2* | 1.0 (Ref) | 0.96 (0.93, 1.00) | 1.12 (1.06, 1.18) | 1.35 (1.21, 1.51) |  | 1.00 (0.99, 1.01) |  | <0.001 |
| HR3† (model 2 + HTN duration) | 1.0 (Ref) | 0.96 (0.93, 1.00) | 1.12 (1.06, 1.18) | 1.35 (1.21, 1.50) |  | 1.00 (0.99, 1.01) |  | <0.001 |

Ref indicates reference. Values in parentheses are 95% CIs.

*Model 2 was adjusted for age, sex, ethnicity, Townsend score, body mass index, smoking status, healthy diet score, diabetes, longstanding illness, hypertensive drug use, and cholesterol-lowering medication use.

†Model 3 was adjusted for variables in model 2 and systolic blood pressure and diastolic blood pressure.

Abbreviations: HTN, hypertension; BP, blood pressure; NA, not applicable.

**Supplementary S12.** Sensitivity analysis for hypertension status and hypertension duration in relation to composite cardiovascular disease.

|  | Without HTN |  | HTN, by duration (y) | | | |
| --- | --- | --- | --- | --- | --- | --- |
|  |  |  | < 5 | ≥ 5 to < 10 | ≥ 10 to < 15 | ≥ 15 |
| Model 2 + CRP | 1.0 (Ref) |  | 1.49 (1.43, 1.56) | 1.57 (1.46, 1.68) | 1.74 (1.60, 1.89) | 2.00 (1.86, 2.16) |
| Model 2 + CRP + CHO | 1.0 (Ref) |  | 1.47 (1.40, 1.53) | 1.55 (1.44, 1.66) | 1.72 (1.59, 1.87) | 1.99 (1.84, 2.14) |
| Model 2 + CRP + CHO + HDL | 1.0 (Ref) |  | 1.48 (1.41, 1.54) | 1.56 (1.45, 1.68) | 1.74 (1.60, 1.88) | 2.00 (1.86, 2.16) |
| Model 2 + CRP + CHO + HDL + physical activity | 1.0 (Ref) |  | 1.48 (1.41, 1.54) | 1.56 (1.45, 1.68) | 1.74 (1.60, 1.89) | 2.00 (1.86, 2.16) |
| Model 2 + age deciles (stratified Cox) | 1.0 (Ref) |  | 1.52 (1.46, 1.59) | 1.60 (1.49, 1.71) | 1.77 (1.63, 1.92) | 2.02 (1.87, 2.17) |
| Exclusion of those with missing data | 1.0 (Ref) |  | 1.52 (1.45, 1.59) | 1.62 (1.50, 1.74) | 1.75 (1.61, 1.74) | 2.01 (1.86, 2.17) |
| Exclusion of those who developed composite CVD in the first 2 years of follow-up | 1.0 (Ref) |  | 1.52 (1.44, 1.59) | 1.61 (1.49, 1.74) | 1.77 (1.61, 1.93) | 1.97 (1.81, 2.14) |
| Exclusion of those with undiagnosed HTN | 1.0 (Ref) |  | 1.57 (1.47, 1.68) | 1.62 (1.50, 1.75) | 1.80 (1.65, 1.96) | 2.04 (1.89, 2.22) |
| Model 3 + CRP | NA |  | 1.0 (Ref) | 1.10 (1.03, 1.17) | 1.22 (1.13, 1.31) | 1.39 (1.30, 1.49) |
| Model 3 + CRP + CHO | NA |  | 1.0 (Ref) | 1.10 (1.03, 1.17) | 1.22 (1.14, 1.32) | 1.41 (1.31, 1.51) |
| Model 3 + CRP + CHO + HDL | NA |  | 1.0 (Ref) | 1.10 (1.03, 1.17) | 1.23 (1.14, 1.32) | 1.40 (1.31, 1.50) |
| Model 3 + CRP + CHO + HDL + physical activity | NA |  | 1.0 (Ref) | 1.10 (1.03, 1.17) | 1.23 (1.14, 1.32) | 1.41 (1.31, 1.50) |
| Model 3 + age deciles (stratified Cox) | NA |  | 1.0 (Ref) | 1.09 (1.03, 1.17) | 1.22 (1.13, 1.31) | 1.38 (1.29, 1.48) |
| Exclusion of those with missing data | NA |  | 1.0 (Ref) | 1.11 (1.04, 1.18) | 1.21 (1.12, 1.30) | 1.37 (1.28, 1.47) |
| Exclusion of those who developed composite CVD in the first 2 years of follow-up | NA |  | 1.0 (Ref) | 1.10 (1.03, 1.18) | 1.21 (1.11, 1.32) | 1.34 (1.25, 1.45) |
| Exclusion of those with undiagnosed HTN | NA |  | 1.0 (Ref) | 1.06 (0.99, 1.14) | 1.18 (1.09, 1.28) | 1.35 (1.25, 1.45) |

Ref indicates reference. Values in parentheses are 95% confidence intervals.

Model 2 was adjusted for age, sex, ethnicity, Townsend score, body mass index, smoking status, healthy diet score, diabetes, longstanding illness, hypertensive drug use, and cholesterol-lowering medication use.

Model 3 was adjusted for age, sex, ethnicity, Townsend score, body mass index, smoking status, healthy diet score, diabetes, longstanding illness, hypertensive drug use, cholesterol-lowering medication use, systolic blood pressure, and diastolic blood pressure.

Abbreviations: HTN, hypertension; BP, blood pressure; CRP, C-reactive protein; CHO, total cholesterol; HDL, high-density lipoprotein; NA, not applicable.

**Supplemental Figure S1. Flowchart of the study population.**

Initial sample from the UK Biobank (n= 502,507)

Exclusion (n= 46,575):

1. Missing on age at HTN diagnosis (n= 14,691);
2. Missing on SBP (n= 1,313);
3. With prevalent CVD (n= 29,271);
4. Lost to follow-up (n= 1,298);
5. Withdrew from the study (n= 2)

Analytical sample (n= 455,932)

Participants without HTN (n= 202,090)

Participants with HTN (n= 253,842)

Abbreviations: CVD, cardiovascular disease; HTN, hypertension

**Supplementary Figure S2**. Systolic blood pressure on a continuous scale and risks of cardiovascular disease and all-cause mortality among participants with hypertension.


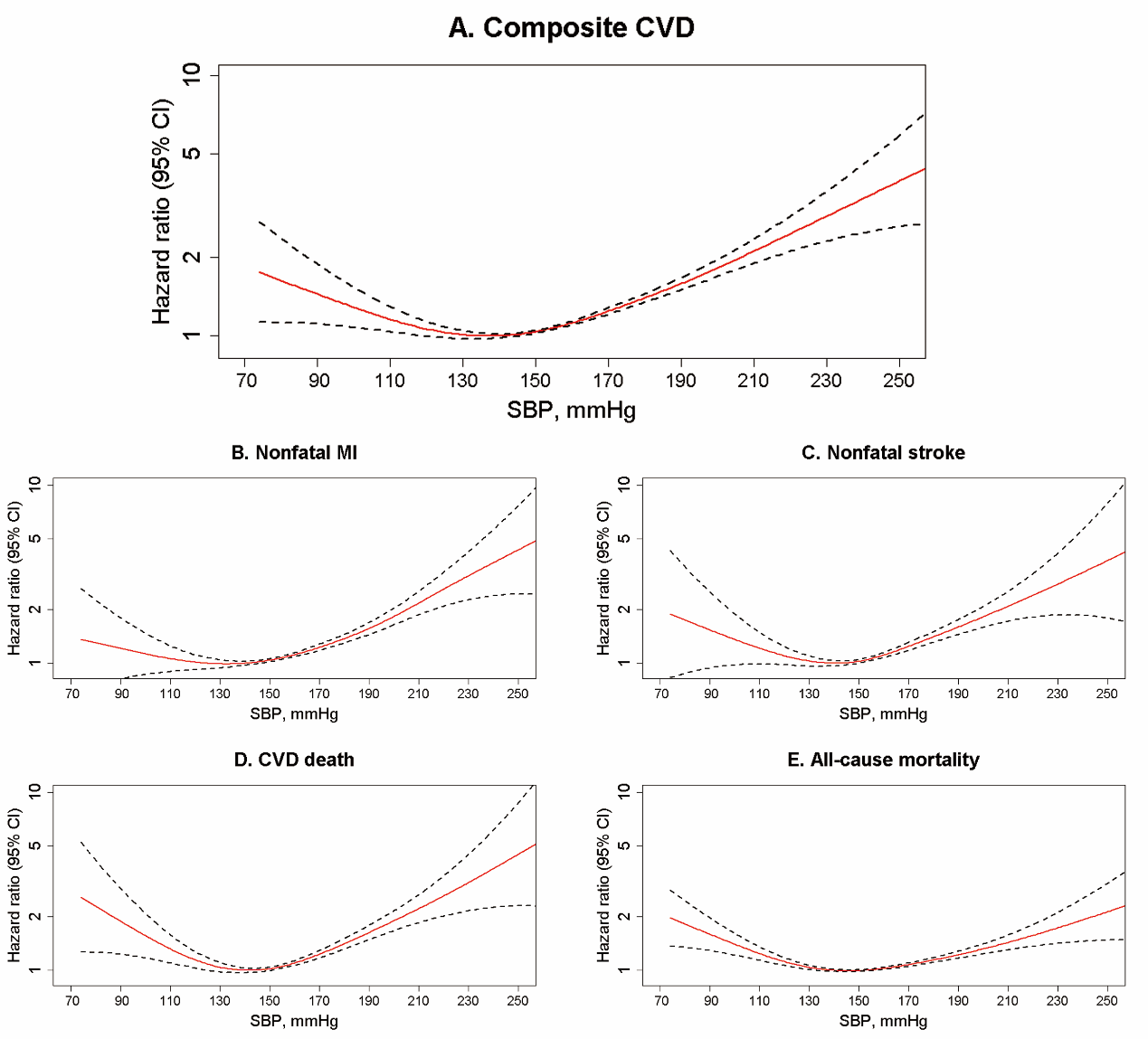


Hazard ratio (solid red line) and 95% confidence interval (dashed black lines) from Cox regression using penalized splines.

Multifactorial adjustments were made for age, sex, ethnicity, Townsend score, body mass index, smoking, healthy diet score, diabetes, longstanding illness, hypertensive drug use, and cholesterol-lowering medication use, and hypertension duration.

Abbreviations: HTN, hypertension; SBP, systolic blood pressure; CVD, cardiovascular disease; MI, myocardial infarction.

**Supplementary Figure S3**. Diastolic blood pressure on a continuous scale and risks of cardiovascular disease and all-cause mortality among participants with hypertension.


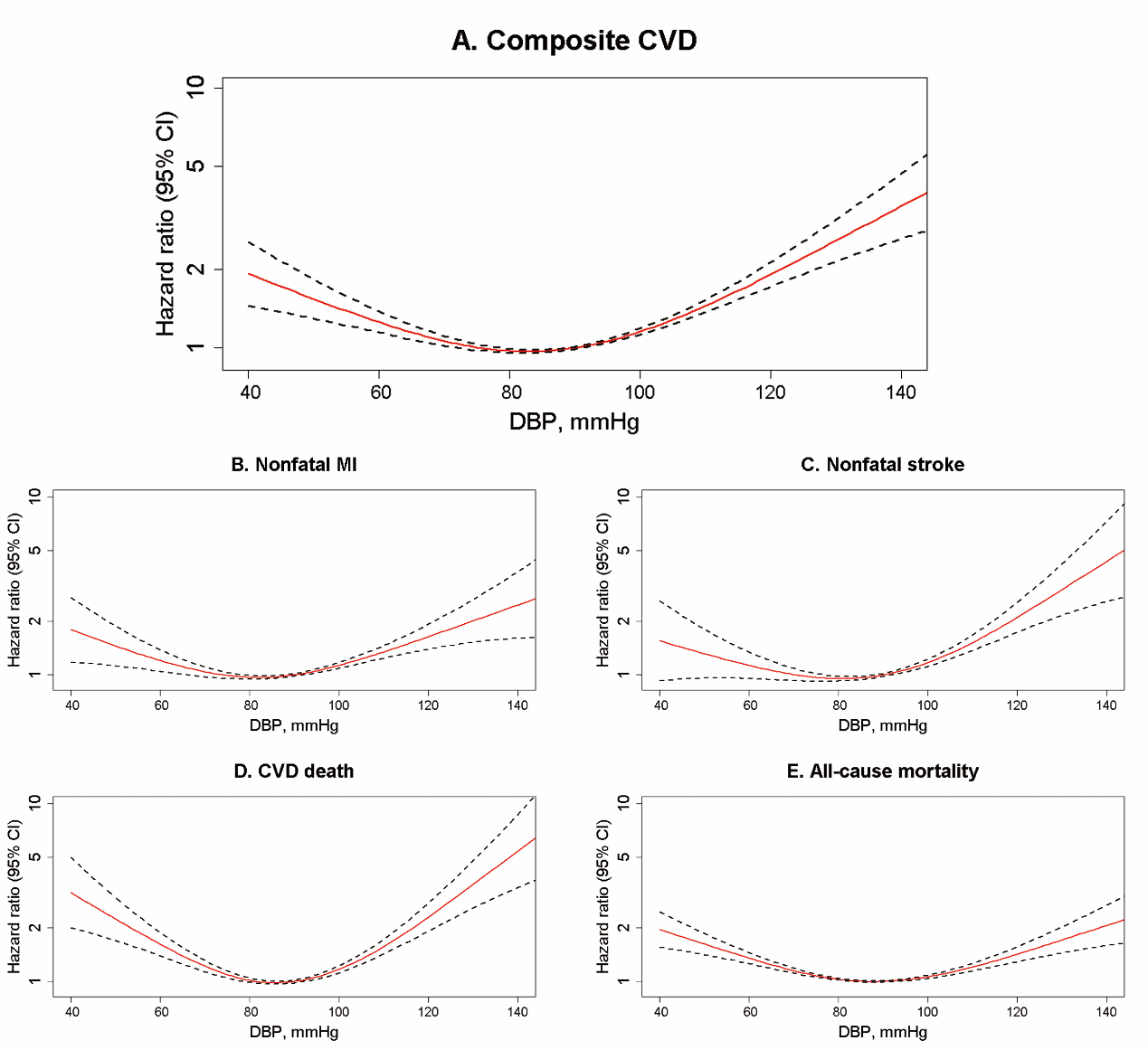


Hazard ratio (solid red line) and 95% confidence interval (dashed black lines) from Cox regression using penalized splines.

Multifactorial adjustments were made for age, sex, ethnicity, Townsend score, body mass index, smoking, healthy diet score, diabetes, longstanding illness, hypertensive drug use, and cholesterol-lowering medication use, and hypertension duration.

Abbreviations: HTN, hypertension; DBP, diastolic blood pressure; CVD, cardiovascular disease; MI, myocardial infarction.

**Supplementary Figure S4.** Cardiovascular disease and all-cause mortality risk matrix for the combined effect of hypotension duration and blood pressure control levels among all participants and participants with hypertension.


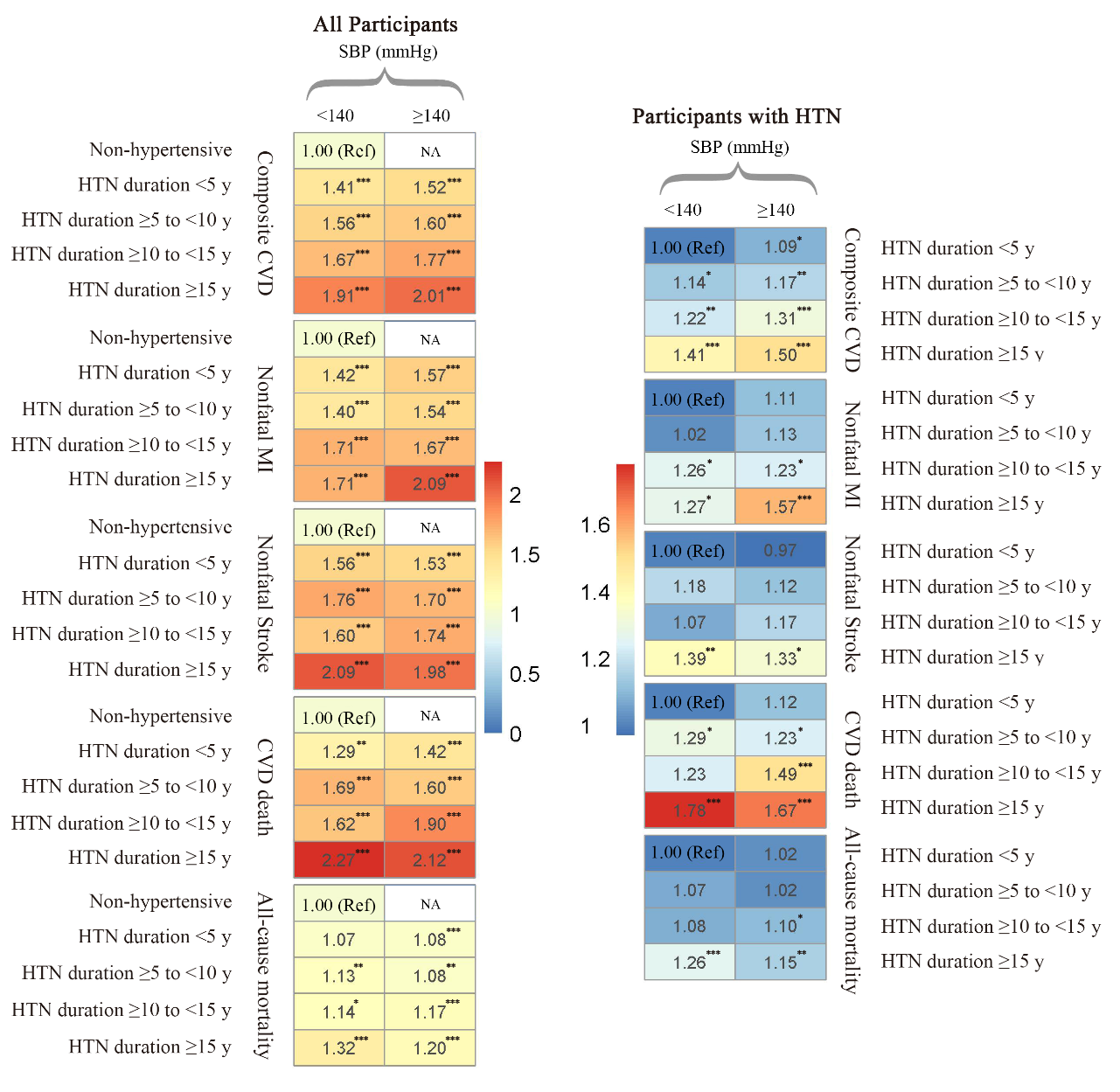


Risk matrixes were developed using Cox models by blood pressure control levels (SBP <140 or ≥140 mmHg). Models were adjusted for age, sex, ethnicity, Townsend score, body mass index, smoking, healthy diet score, diabetes, longstanding illness, hypertensive drug use, and cholesterol-lowering medication use.

Abbreviations: HTN, hypertension; CVD, cardiovascular disease; SBP, systolic blood pressure; MI, myocardial infarction; Ref, reference; NA, not applicable.

****P*<0.001; ***P*<0.01; **P*<0.05.

**Supplementary Figure S5.** Cardiovascular disease and all-cause mortality risk matrix for the combined effect of hypotension duration and blood pressure control levels among all participants and participants with hypertension.


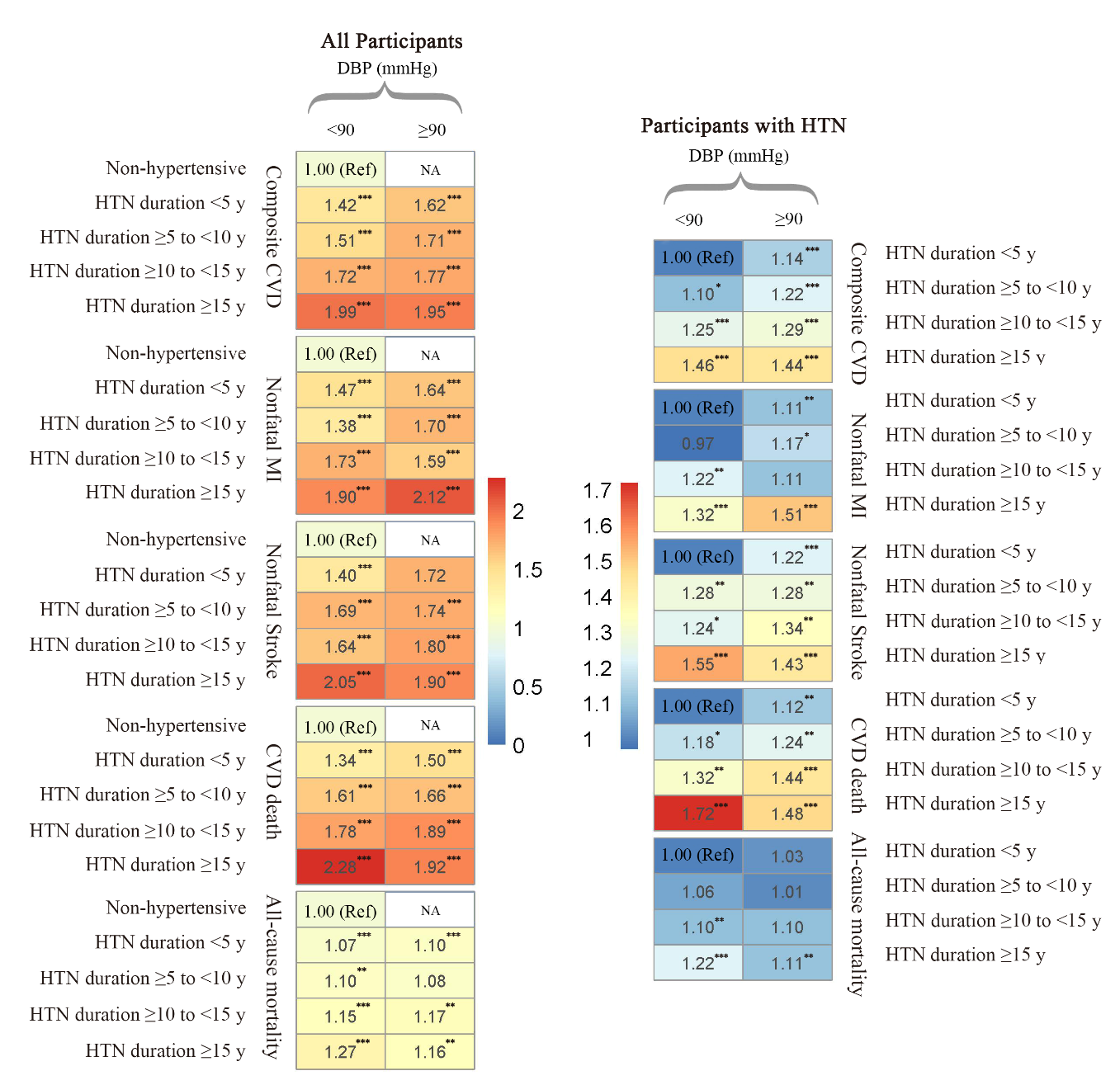


Risk matrixes were developed using Cox models by blood pressure control levels (DBP <90 or ≥90 mmHg). Models were adjusted for age, sex, ethnicity, Townsend score, body mass index, smoking, healthy diet score, diabetes, longstanding illness, hypertensive drug use, and cholesterol-lowering medication use.

Abbreviations: HTN, hypertension; CVD, cardiovascular disease; DBP, diastolic blood pressure; MI, myocardial infarction; Ref, reference; NA, not applicable.

****P*<0.001; ***P*<0.01; **P*<0.05.

**Supplementary Figure S6.** Hypertension duration on a continuous scale and risk of composite cardiovascular disease among participants with hypertension by different subgroups.


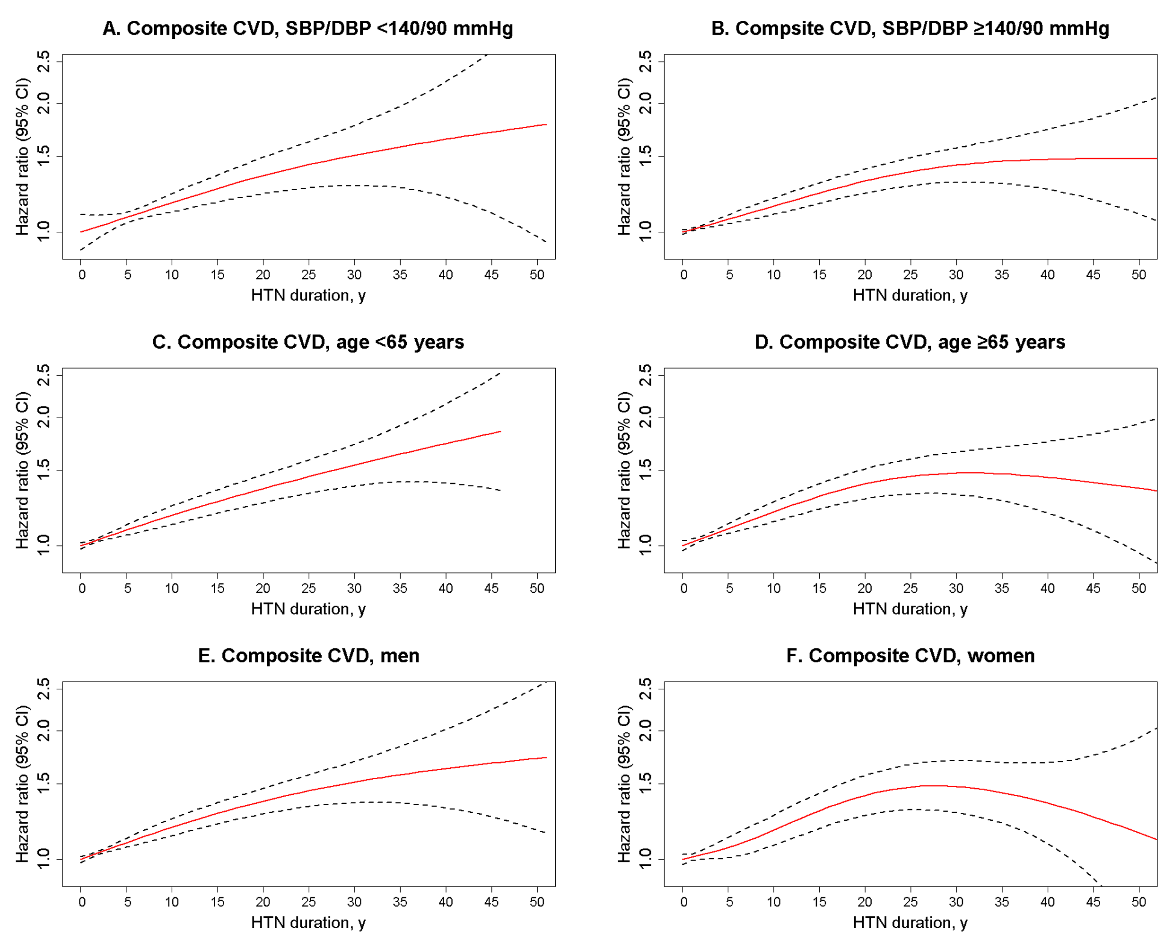


Hazard ratio (solid red line) and 95% confidence interval (dashed black lines) from Cox regression using penalized splines.

Multifactorial adjustments were made for age, sex, ethnicity, Townsend score, body mass index, smoking, healthy diet score, diabetes, longstanding illness, hypertensive drug use, cholesterol-lowering medication use, systolic blood pressure and diastolic blood pressure.

Abbreviations: HTN, hypertension; CVD, cardiovascular disease; MI, myocardial infarction.

**Supplementary Figure S7.** Systolic blood pressure on a continuous scale and risk of composite cardiovascular disease among participants with hypertension by different subgroups.


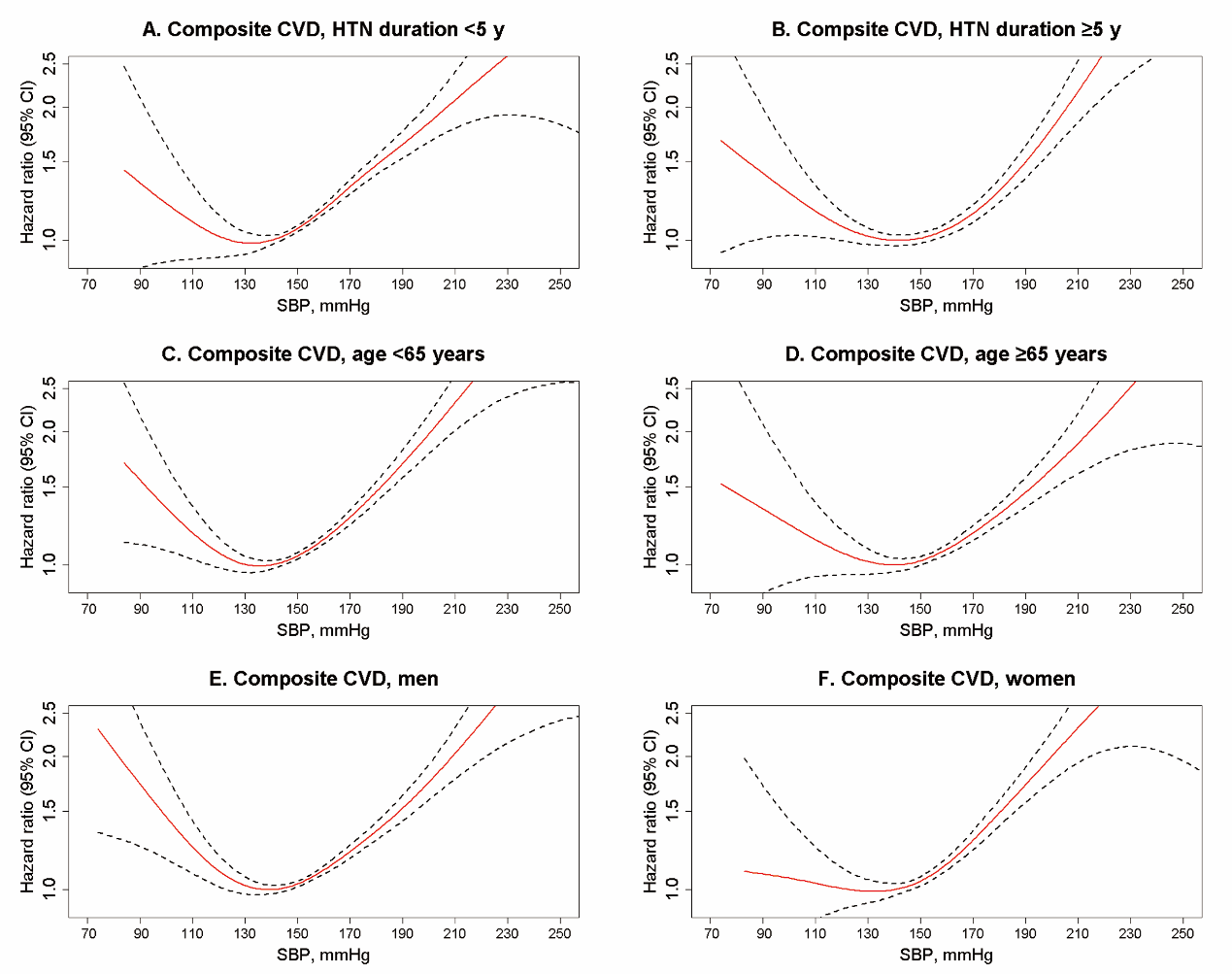


Hazard ratio (solid red line) and 95% confidence interval (dashed black lines) from Cox regression using penalized splines.

Multifactorial adjustments were made for age, sex, ethnicity, Townsend score, body mass index, smoking, healthy diet score, diabetes, longstanding illness, hypertensive drug use, cholesterol-lowering medication use, and hypertension duration.

Abbreviations: HTN, hypertension; CVD, cardiovascular disease; SBP, systolic blood pressure; MI, myocardial infarction.

**Supplementary Figure S8.** Diastolic blood pressure on a continuous scale and risk of composite cardiovascular disease among participants with hypertension by different subgroups.


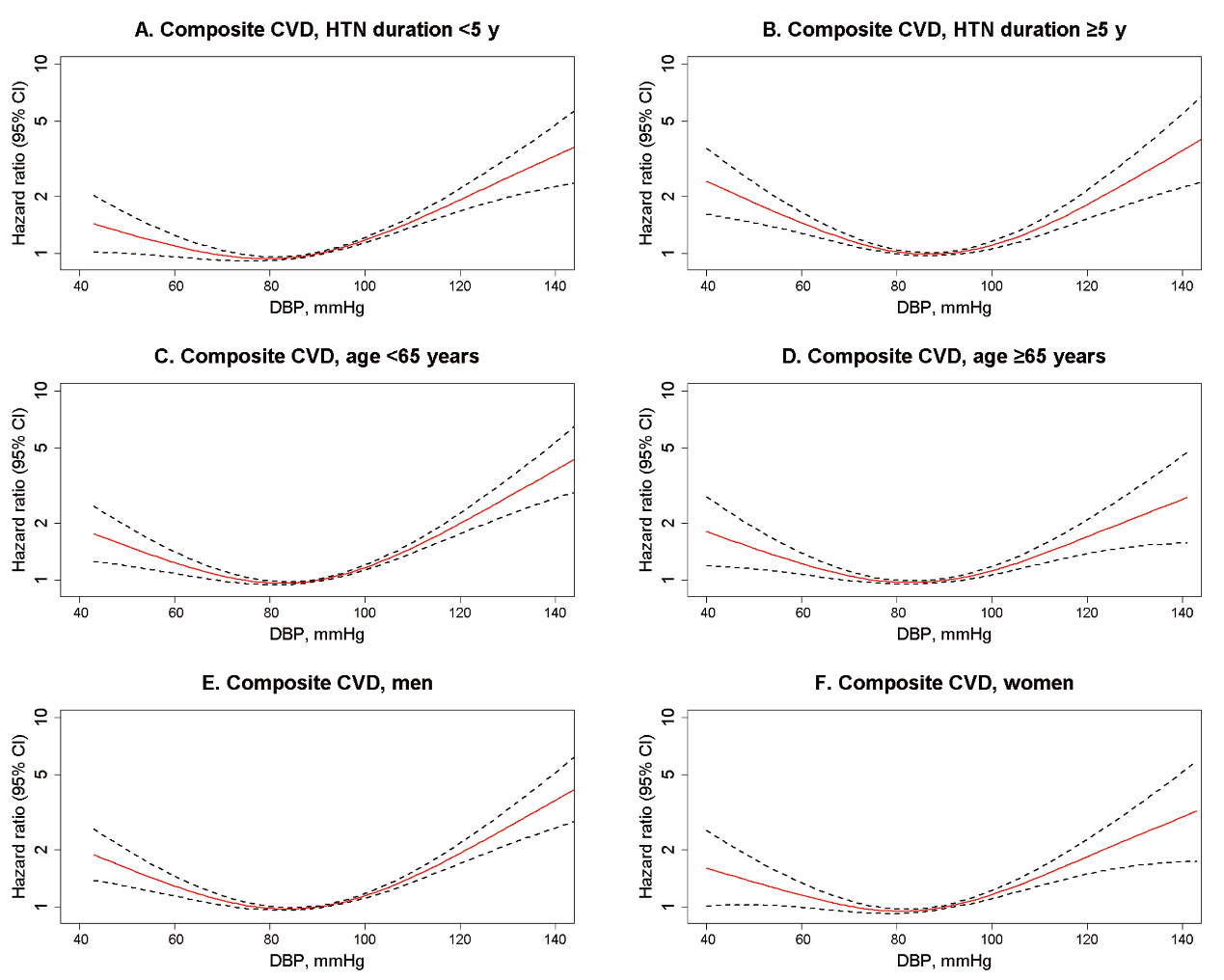


Hazard ratio (solid red line) and 95% confidence interval (dashed black lines) from Cox regression using penalized splines.

Multifactorial adjustments were made for age, sex, ethnicity, Townsend score, body mass index, smoking, healthy diet score, diabetes, longstanding illness, hypertensive drug use, cholesterol-lowering medication use, and hypertension duration.

Abbreviations: HTN, hypertension; CVD, cardiovascular disease; DBP, diastolic blood pressure; MI, myocardial infarction.
